# Development of selective inhibitors of phosphatidylinositol 3-kinase C2α

**DOI:** 10.1038/s41589-022-01118-z

**Published:** 2022-09-15

**Authors:** Wen-Ting Lo, Hassane Belabed, Murat Kücükdisli, Juliane Metag, Yvette Roske, Polina Prokofeva, Yohei Ohashi, André Horatscheck, Davide Cirillo, Michael Krauss, Christopher Schmied, Martin Neuenschwander, Jens Peter von Kries, Guillaume Médard, Bernhard Kuster, Olga Perisic, Roger L. Williams, Oliver Daumke, Bernard Payrastre, Sonia Severin, Marc Nazaré, Volker Haucke

**Affiliations:** 1 Leibniz-Forschungsinstitut für Molekulare Pharmakologie (FMP), Berlin, Germany; 2 Max-Delbrück-Centrum für Molekulare Medizin, Kristallographie, Berlin, Germany; 3 Chair of Proteomics and Bioanalytics, Technical University of Munich, Freising, Germany; 4 MRC Laboratory of Molecular Biology, Cambridge Biomedical Campus, Cambridge, UK; 5 Inserm, U1297-Université, Toulouse III, Institut des Maladies Métaboliques et Cardiovasculaires, Toulouse, France; 6 Centre Hospitalier Universitaire de Toulouse, Laboratoire d’Hématologie, Toulouse, France; 7 Departments of Biology, Chemistry, and Pharmacy, Freie Universität Berlin, Berlin, Germany

**Keywords:** Small molecules, X-ray crystallography, Membrane trafficking

## Abstract

Phosphatidylinositol 3-kinase type 2α (PI3KC2α) and related class II PI3K isoforms are of increasing biomedical interest because of their crucial roles in endocytic membrane dynamics, cell division and signaling, angiogenesis, and platelet morphology and function. Herein we report the development and characterization of PhosphatidylInositol Three-kinase Class twO INhibitors (PITCOINs), potent and highly selective small-molecule inhibitors of PI3KC2α catalytic activity. PITCOIN compounds exhibit strong selectivity toward PI3KC2α due to their unique mode of interaction with the ATP-binding site of the enzyme. We demonstrate that acute inhibition of PI3KC2α-mediated synthesis of phosphatidylinositol 3-phosphates by PITCOINs impairs endocytic membrane dynamics and membrane remodeling during platelet-dependent thrombus formation. PITCOINs are potent and selective cell-permeable inhibitors of PI3KC2α function with potential biomedical applications ranging from thrombosis to diabetes and cancer.

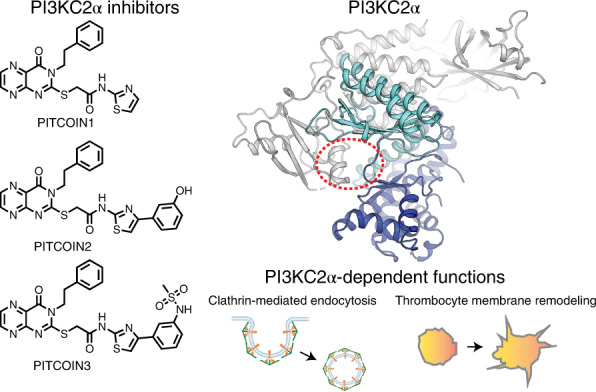

## Abstract (web summary)

Compound library screening combined with medicinal chemistry and structural biology approaches enables the development of potent and highly selective cell-permeable small-molecule inhibitors of phosphatidylinositol 3-kinase C2α activity and function.

## Main

Phosphoinositide 3-kinases (PI3Ks) are a family of ubiquitously distributed lipid kinases that phosphorylate inositol lipids at the 3′-OH group to regulate cell and organismal physiology. Mammals including humans express three distinct classes of PI3Ks, consistent with the large repertoire of processes that are controlled by phosphatidylinositol (PI) 3-phosphates^1–3^. Heterodimeric class I PI3Ks signal downstream of plasma membrane-bound receptors and small GTPases (for example, Ras) via synthesis of PI 3,4,5-trisphosphate (PI(3,4,5)P_3_) ^2,4–6^ to regulate metabolism, proliferation and migration. Aberrant class I PI3K signaling has a prominent role in cancer and immunity and, hence, isoform-selective as well as pan-class I PI3K inhibitors have been the focus of extensive drug development efforts and hold promise as anticancer therapeutics among other biomedical applications^1,7^. Vps34, the sole class III PI3K member, is present in two related protein complexes that synthesize PI 3-monophosphate (PI(3)P) within the endolysosomal system and during autophagy as well as in cytokinesis^1^. The recent development of selective Vps34 inhibitors^8,9^ has greatly facilitated the analysis of class III PI3K function in biology and may be of potential clinical use in cancer and other disorders related to autophagy or rare genetic diseases caused by imbalance of PI 3-phosphate levels (for example, Charcot–Marie–Tooth disease type 4B)^10,11^.

Class II PI3Ks comprise PI3KC2α, PI3KC2β and PI3KC2γ isoforms and are unique in their ability to directly synthesize PI 3,4-bisphosphate (PI(3,4)P_2_) from PI 4-monophosphate (PI(4)P) in vivo. In addition, they have been suggested to contribute to cellular PI(3)P production on endosomes and in the autophagy/lysosomal pathway^7,12–14^. PI3KC2α and PI3KC2β are ubiquitously expressed, whereas PI3KC2γ is mostly expressed in exocrine glands and liver. PI3KC2α is encoded by an essential gene in mice^14^ and has been implicated in a variety of cellular and organismal functions. On a cellular level, PI3KC2α controls clathrin-mediated endocytic vesicle formation^15,16^ and internalization of vascular endothelial growth factor (VEGF)^17^ and transforming growth factor β receptors^18,19^, endocytic recycling^13^, cytokinesis^20^, primary cilia function^14^ and insulin signaling^21^. Accumulating evidence further implicates PI3KC2α in cancer^1,22,23^ and cataract formation^20^, regulation of blood pressure^24^, viral replication^25,26^, angiogenesis^17^ and platelet-dependent thrombus formation as well as membrane shear-dependent platelet adhesion^27–29^. In spite of its importance for cell and organismal physiology and the potential biomedical use of PI3KC2α inhibitors, for example, as antithrombotic, antidiabetic or antiangiogenic drugs, no isoform-selective inhibitors of PI3KC2α catalytic activity are available.

Here we report on the development and characterization of PhosphatidylInositol Three-kinase Class twO INhibitors (PITCOINs) 1–3 (compounds **1**–**3**), potent and highly selective cell-permeable small-molecule inhibitors of PI3KC2α activity and function. High-resolution co-crystal structures of PI3KC2α in complex with PITCOIN inhibitors reveal a unique mode of interaction with the adenosine triphosphate (ATP)-binding site that provides a rationale for the exclusive selectivity of these inhibitors for PI3KC2α with no off-target activity toward other lipid and protein kinases. PITCOINs impair PI3KC2α-mediated synthesis of PI(3,4)P_2_ and PI(3)P at plasma membrane endocytic nanostructures and on endosomes, respectively, and exhibit potent antithrombotic activity by counteracting platelet membrane remodeling. PITCOINs may thus serve as a starting point for the development of drugs targeting class II PI3K function for therapeutic applications.

## Results

### PITCOINs potently and selectively inhibit PI3KC2α activity

**Fig. 1:**
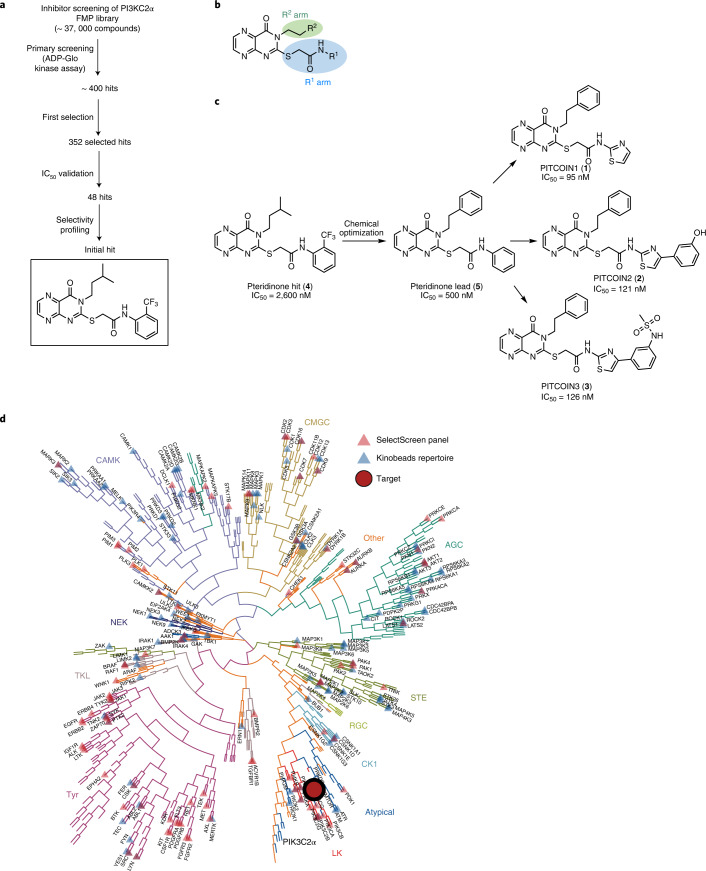
PITCOINs potently and selectively inhibit PI3KC2α activity. **a**, Scheme of inhibitor screening and development. The structure of the initial lead compound with an IC_50_ of 2.6 µM is boxed. **b**, Schematic representation of the synthetic strategy used to generate focused libraries of pteridinone-containing molecules in which the R^1^ and R^2^ groups are varied. **c**, Chemical structures of PITCOIN1–PITCOIN3. The molecules share a pteridinone scaffold and a terminal phenyl group on the R^1^ arm but harbor distinct thiazole-substituted R^2^ moieties. **d**, Kinome tree representation of kinase selectivity profiling of PITCOIN3. PITCOIN3 is highly selective in targeting PI3KC2α but no other human kinases. Source data

**Extended Data Fig. 1:**
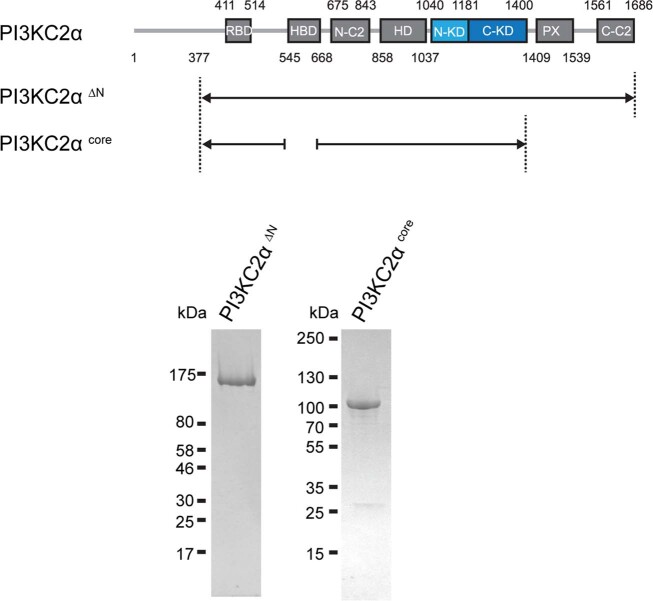
Domain architecture and purity of recombinant human PI3KC2α^∆N^ and mouse PI3KC2α^core^. Domain architecture of PI3KC2α and Coomassie Blue-stained SDS-PAGE analysis of purified recombinant human PI3KC2α^∆N^ and mouse PI3KC2α^core^. Both proteins are > 90% pure (n = 2 individual purification batches). Purified human PI3KC2α^∆N^ was used for inhibitor development, whereas mouse PI3KC2α^core^ was used for protein crystallization. Source data

While PI3KC2α is partly refractory to inhibition by wortmannin, it can be targeted by a subset of nonselective PI3K inhibitors such as Torin 2 or PIK-90 and its derivatives^4,28,30^ that are promiscuous with respect to their cellular activities. We therefore reasoned that a distinct chemical scaffold may be required to selectively target PI3KC2α function. To identify such new chemical scaffolds, we conducted high-throughput screening (HTS) of in-house chemical libraries using purified recombinant PI3KC2α (Extended Data Fig. 1) followed by iterative rounds of medicinal chemistry optimization (Fig. 1a). We used an adenosine diphosphate (ADP)-Glo assay to screen about 37,000 compounds for their ability to inhibit PI3KC2α activity using PI as a substrate and determined the IC_50_ values of 352 initial hits (Supplementary Table 1). Further selectivity profiling of 48 of these hits against a panel of lipid kinases resulted in the identification of a PI3KC2α-selective inhibitor (**4**) containing a pteridinone scaffold that displayed a moderate half-maximal inhibitory concentration (IC_50_) of 2.6 µM (Fig. 1a). This pteridinone hit compound, a so far underexplored chemotype for kinase inhibition, served as a lead structure for further optimization by medicinal chemistry approaches (Supplementary Note). We focused on substitutions of the terminal groups on the R^1^ and R^2^ arms of the pteridinone scaffold (Fig. 1b). Modifications by removal of the *ortho*-trifluoromethyl group in R^1^ and replacement of the isopropyl group in R^2^ with a phenyl moiety improved the potency about fivefold for inhibitor (**5**; IC_50_ (PI3KC2α) of 0.5 µM). Further optimization focused on replacement of the terminal R^1^ group. This strategy gave rise to three thiazole-substituted molecules with nanomolar IC_50_ values and high selectivity toward PI3KC2α that we refer to as PITCOIN1–PITCOIN3 (Fig. 1c). While PITCOIN1 carries a plain thiazole ring, the PITCOIN2 and PITCOIN3 congeners are extended at the 4′ position with an additional meta-substituted phenyl ring either by a phenolic hydroxy group or by a bioisosteric *N*-methanesulfonamide. These compounds inhibited PI3KC2α to a similar extent with IC_50_ values between 95 and 126 nM but differed with respect to their effects on the closely related class II PI3K isoforms PI3KC2β and PI3KC2γ. While PITCOIN1 was found to be a moderate PI3KC2γ and PI3KC2β inhibitor with IC_50_ values of about 1 µM or above, PITCOIN2 exhibited selectivity against PI3KC2γ but showed micromolar activity on PI3KC2β. In contrast, PITCOIN3 displayed exquisite specificity for PI3KC2α with no detectable interference with PI3KC2β and PI3KC2γ up to concentrations of 10 µM (Supplementary Table 2).

**Extended Data Fig. 2:**
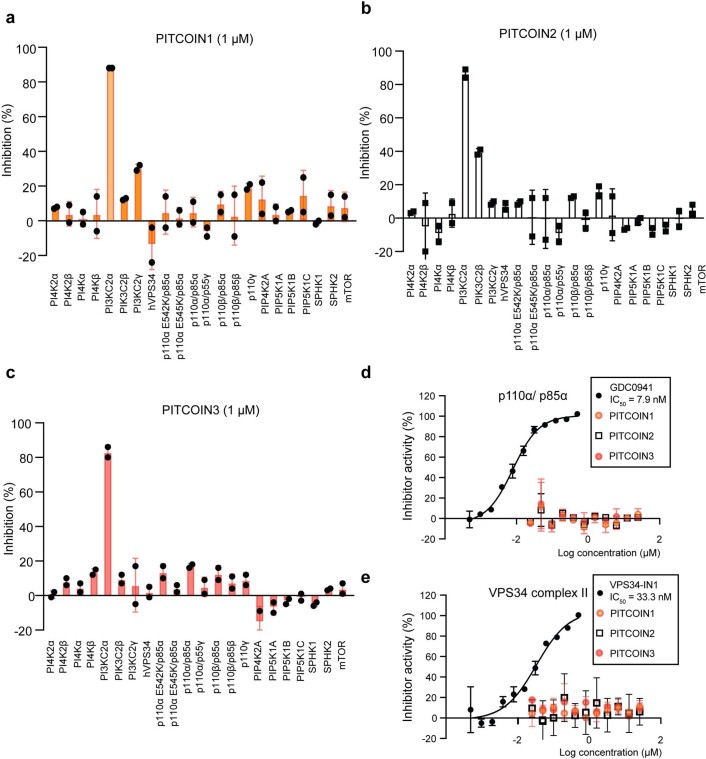
Lipid kinase inhibitor profile of PITCOIN1-3. (**a**–**c**) Selectivity profiling of PITCOIN1-3 (1 µM) with purified lipid kinases in the presence of 10 µM ATP. PITCOIN1-3 are highly selective for PI3KC2α. Data are presented as mean ± SD from two independent experiments. (**d**, **e**) IC_50_ measurements of PITCOIN1-3 against purified recombinant class I PI3K p110α/p85α (**d**) or purified class III PI3K VPS34 complex II (**e**). 10 µM ATP and 20 µM PIP_3_ (for p110α/p85α) or 200 µM PI (for VPS34 complex II) were used as substrates. The class I PI3K inhibitor GDC0941 or VPS34-IN1 were used as positive controls. PITCOIN1-3 were inactive towards p110α/p85α and VPS34 complex II up to 20 µM. Data are presented as mean ± SD from two independent experiments. Source data

As PI3KC2α is related to other PI3Ks and to PI3K-like protein kinases such as mTOR, the selectivity of PITCOINs was determined in a panel of biochemical assays. Selectivity profiling revealed no off-target activity of PITCOIN1–PITCOIN3 toward a panel of 117 purified kinases including related lipid kinases and mTOR (Extended Data Fig. 2a,b and Supplementary Dataset). Notably, PITCOIN1–PITCOIN3 were found to be inactive against class I PI3Kα or purified Vps34 complex II (Extended Data Fig. 2c–e). Finally, the high specificity of PITCOINs for PI3KC2α was verified by Kinobead profiling experiments. In this approach, the ability of PITCOINs to compete the affinity capture of cellular kinases on immobilized nonselective kinase inhibitors (Kinobeads) is analyzed by mass spectrometry (MS)^31^. Incubation of HEK293T cell lysates with increasing concentrations (30 nM to 1 mM) of PITCOIN1–PITCOIN3 resulted in the selective, robust competition of solely PI3KC2α and no other among the 137 quantified kinases in the Kinobead profiling assay (Fig. 1d). Four nonkinase weak off-targets were globally found for the three inhibitors with only FECH^32^ common to the three PITCOINs (Supplementary Dataset).

These results identify PITCOINs as selective inhibitors of PI3KC2α and related class II PI3Ks with PITCOIN3 exclusively targeting the PI3KC2α isoform.

### Structural basis for PI3KC2α inhibition by PITCOINs

**Fig. 2:**
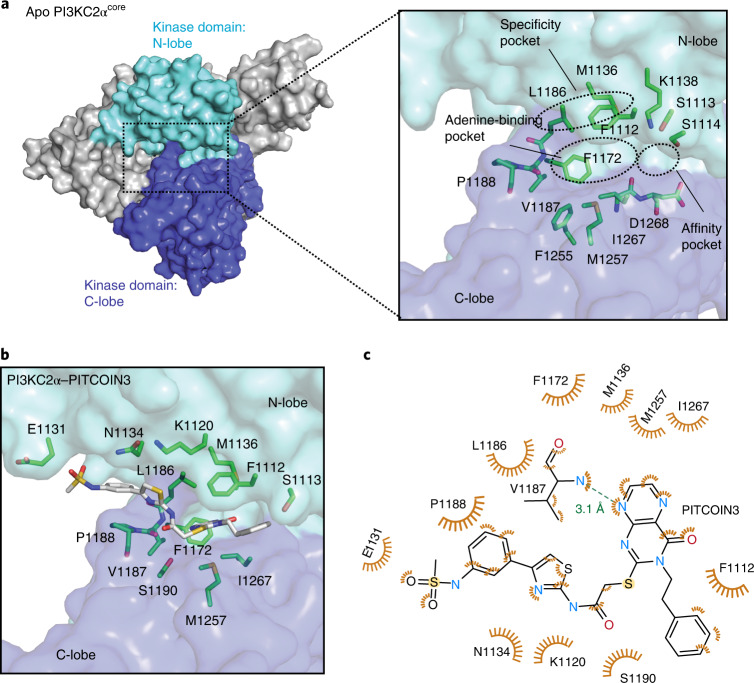
Structural basis for PI3KC2α inhibition by PITCOINs. **a**, Surface representation of the overall structure of apo PI3KC2α^core^. The N- and C-lobes of the kinase domain are highlighted in cyan and blue, respectively. Inset, close-up view of the ATP-binding pocket. The adenine-binding pocket, affinity pocket and specific pocket are indicated by dashed circles. Key residues are shown by stick representation. Protein Data Bank (PDB) code: 7BI4. **b**, PI3KC2α^core^ in complex with PITCOIN3. The *N*-phenylmethanesulfonamide on the 4′ position of the thiazole moiety is rotated by 180° compared to the conformation in PITCOIN2. **c**, Schematic representation of interactions between PITCOIN3 and PI3KC2α. Hydrophobic interactions are shown as orange eyelashes. A unique hydrogen bond is shown as a green dashed line with the distance between the donor and acceptor atoms indicated. Hydrogen atoms were omitted for clarity. The phenyl sulfonamide group displays hydrophobic interactions with P1188 and E1131.

To unravel the structural determinants of inhibitor potency and specificity for PI3KC2α, we capitalized on the recent determination of high-resolution structures of PI3KC2α in its active and inactive conformations by cryogenic electron microscopy (cryo-EM) and protein X-ray crystallography^30^. PI3KC2α contains a compact PI3K catalytic core (PI3KC2α^core^) that comprises a Ras-binding domain and an N-terminal C2 domain as well as helical and kinase domains and displays a typical PI3K kinase domain fold^30^. The ATP-binding site of the unliganded apo form of PI3KC2α^core^ is located in a cavity between the N- and C-lobes of the kinase domain (Fig. 2a) and encompasses the (1) adenine-binding pocket, (2) affinity pocket and (3) specificity pocket. The adenine-binding pocket consists of a hinge region (L1186, V1187 and P1188) that connects the N- and C- lobes of the kinase domain, a hydrophobic region (F1255, M1257 and I1267) that forms the mouth of the pocket and a conserved aromatic residue (F1172). The small affinity pocket harbors conserved hydrogen-bond donor or acceptor residues (for example, K1138 and D1268). The specificity pocket comprises P-loop residues (F1112 and S1113) and the PI3KC2α-unique residue M1136 (Fig. 2a, inset). A notable feature of the ATP-binding site of PI3KC2α is that L1186 within the kinase hinge is tightly packed against the P-loop residue F1112, which might rigidify its conformation.

**Extended Data Fig. 3:**
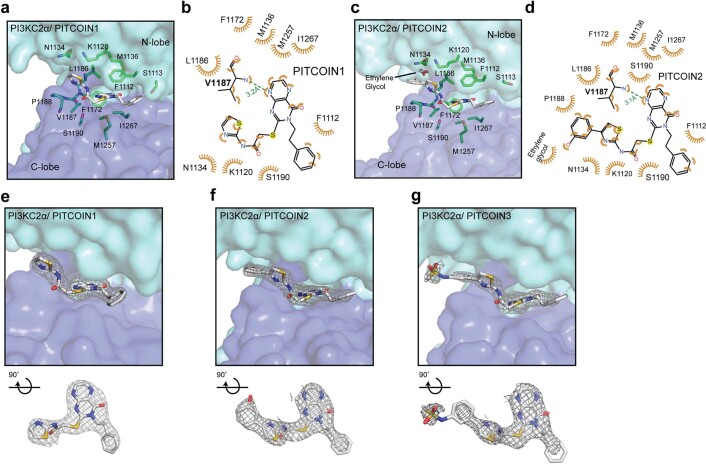
Structural basis for PITCOIN selectivity. (**a**) PI3KC2α^core^ in complex with PITCOIN1. PITCOIN1 displays a propeller-shaped conformation in the ATP binding site. The vicinal R^1^ and R^2^ arms extend outward from the adenine-binding pocket and interact with specific residues in the N-lobe of the kinase domain. (**b**) Schematic representation of interactions between PITCOIN1 and PI3KC2α. Hydrophobic interactions are shown as orange eyelashes. A unique hydrogen bond is shown as a green dashed line with the distance between donor and acceptor atom indicated. (**c**) PI3KC2α^core^ in complex with PITCOIN2. The *N-*phenylmethanesulfonamide on the 4′-position of the thiazole moiety is stabilized by interaction with the solvent. (**d**) Schematic representation of interactions between PITCOIN2 and PI3KC2α. Hydrophobic interactions are shown as orange eyelashes. A unique hydrogen bond is shown as a green dashed line with the distance between donor and acceptor atom indicated. (**e**–**g**) Electron density maps of PITCOIN1 (**e**), PITCOIN2 (**f**), and PITCOIN3 (**g**) in complex with mouse PI3KC2α^core^. Top: PITCOIN1-3 occupy the ATP binding pocket of PI3KC2α. The 2Fo-Fc maps of PITCOIN1-3 are illustrated as gray meshes at a sigma level of 1.0. Bottom: 90°-rotated 2Fo-Fc maps of PITCOIN1-3 viewed from the top. PI3KC2α^core^ has been omitted for clarity.

To explore the structural basis of the inhibitory mechanism of PITCOIN1–PITCOIN3, we determined the structures of PI3KC2α^core^ in complex with inhibitors at maximal resolutions ranging from 2.5 to 2.9 Å (Fig. 2b,c, Extended Data Fig. 3a–g and Supplementary Table 3). PITCOINs adopt nearly identical positions in the ATP-binding site. Their pteridinone scaffold occupies the adenine-binding pocket, while the two vicinal (R^1^ and R^2^) arms that extend outward from the pteridinone moiety display specific interactions with the N-lobe of the kinase domain and cause the inhibitors to adopt a propeller-shaped conformation. The affinity pocket remains empty. The pteridinone scaffold of PITCOIN1–PITCOIN3 forms a single hydrogen bond with the backbone of V1187 located in the kinase hinge region and embeds into hydrophobic surfaces provided by M1136 and L1186 at the top, M1257 and I1267 at the bottom, and F1172 on the inside of the adenine-binding pocket. The terminal phenyl group that forms the R^2^ arm binds to F1112 on the P-loop via hydrophobic interactions (Fig. 2b,c and Extended Data Fig. 3a–d). The importance of the hydrophobic R^2^ interactions is underscored by the fact that substitution of the isopropyl group in the initial hit compound 4 by a phenyl moiety led to considerably increased inhibitory potency (that is, a >5-fold-improved IC_50_; Fig. 1c). The R^1^ arm displays an L-shaped conformation, in which one edge contacts S1190 within the C-lobe, while the thiazole group is placed into a hydrophobic pocket formed by L1186 within the kinase hinge and the N-lobe residues K1120 and N1134 (Fig. 2c and Extended Data Fig. 3b,d), that is, a site that has not been identified previously as an inhibitor target site in other PI3Ks.

PITCOIN2 and PITCOIN3 are distinguished from PITCOIN1 by the presence of bulky hydroxyphenyl (that is, PITCOIN2) or *N*-phenylmethanesulfonamide (that is, PITCOIN3) substituents on the 4′ position of the thiazole group. The space demand of these polar and bulky groups leads to additional hydrophobic interaction between P1188 within the kinase hinge and the phenyl group on the R^1^ arm of PITCOIN2 and PITCOIN3. As a result of these alterations, the mild off-target activity of PITCOIN1 against PI3KC2γ is lost in PITCOIN2 and PITCOIN3. Interestingly, the terminal hydroxyphenyl and *N*-phenylmethanesulfonamide groups cause the respective phenyl moiety to adopt different orientations upon complex formation of PITCOIN2 and PITCOIN3 with PI3KC2α. The hydroxyphenyl group of PITCOIN2 faces inward and is stabilized through interaction with the solvent, whereas the *N-*phenylmethanesulfonamide group of PITCOIN3 forms hydrophobic interactions with E1131 (Fig. 2b,c and Extended Data Fig. 3c,d).

**Extended Data Fig. 4:**
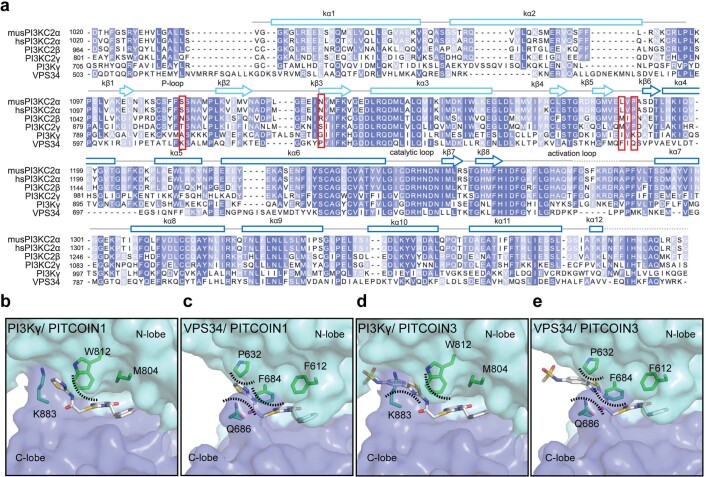
PITCOINs do not fit the ATP binding pockets of PI3Kγ or VPS34. (**a**) Multiple sequence alignment of PI3K kinase domains using mouse PI3KC2α (musPI3KC2α) as a template. Hs, *Homo sapiens*, mus, *Mus musculus*. The following sequences were aligned: human PI3KC2α (hsPI3KC2α, UniProt accession: O00443.2), human PI3KC2β (UniProt accession: O00750.2), human PI3KC2γ (UniProt accession: O75747.3)], human class I PI3K (PI3Kγ, UniProt accession: P48736.3), and human class III PI3K (VPS34, UniProt accession: Q8NEB9.1). Amino acids S1113, N1134, L1186, and P1189 of musPI3KC2α that are crucial for inhibitor selectivity are marked by a red square. (**b**-**e**) Docking model for PITCOIN1 or PITCOIN3 in the ATP binding pockets of PI3Kγ (**b** and **c**) or VPS34 (**d** and **e**). PITCOINs were docked by superimposition of the kinase domain of PI3KC2α with those of PI3Kγ or VPS34. Dashed lines indicate steric clashes. Source data

Interestingly, many of the target residues of the protein backbone contacted by the R^1^ and R^2^ arms of PITCOIN1–PITCOIN3 are not conserved in other class II PI3K isoforms, for example, N1134 (PI3KC2β: R1079, PI3KC2γ: S916), L1186 (PI3KC2β: M1131, PI3KC2γ: M968) and S1113 (PI3KC2β: N1058, PI3KC2γ: T895) (Extended Data Fig. 4a). Together with the different lengths and space requirements of the R^1^ arms of PITCOIN1 versus PITCOIN2 or PITCOIN3, these features provide a structural explanation for the high degree of selectivity of PITCOINs for PI3KC2α over other class II PI3K isoforms and further members of the PI3K family. Consistent with this, we observe that placement of PITCOIN1 (Extended Data Fig. 4b,c) or PITCOIN3 (Extended Data Fig. 4d,e) into the ATP-binding pockets of class I PI3Kγ or Vps34 results in a steric clash of the R^1^ arm and the thiazole phenyl group of PITCOIN3 with residues in the ATP-binding site.

Collectively, these results from structural–biochemical analysis of the protein–ligand interaction provide a molecular explanation for the potency and specificity of PITCOINs for PI3KC2α.

### PITCOINs impair PI(3,4)P_2_ synthesis and endocytosis

**Fig. 3:**
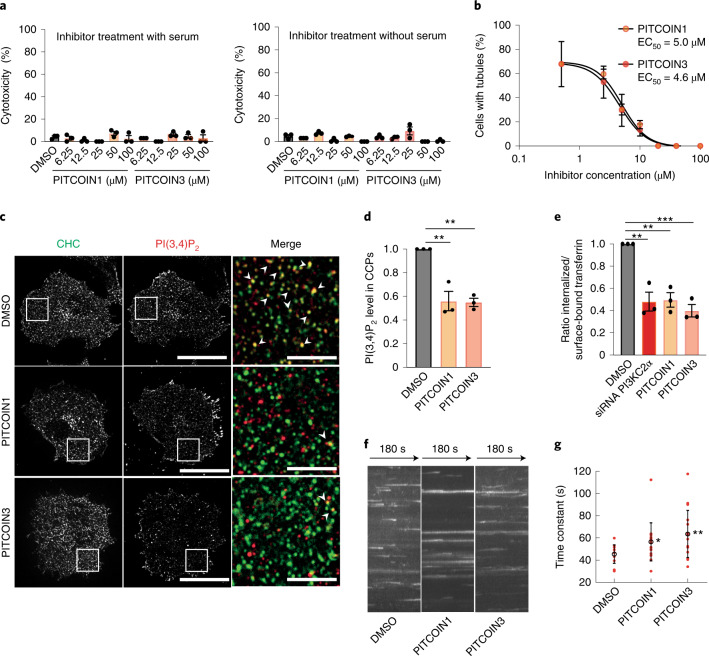
PITCOINs impair PI(3,4)P_2_ synthesis and endocytosis. **a**, PITCOIN1 and PITCOIN3 are nontoxic. HeLa cells treated for 20 h with the indicated concentrations of PITCOIN1 and PITCOIN3 were subjected to trypan blue uptake assays. Mean ± s.e.m.; *n* = 3. **b**, Dose-dependent block of plasma membrane tubulation induced by eGFP–SNX9. HeLa cells expressing constitutively active eGFP–SNX9 were treated with PITCOIN1 or PITCOIN3 (6 h) and the fraction of cells with tubules was quantified. Mean ± s.e.m.; *n* = 3. **c,d**, Inhibition of PI3KC2α inhibits local PI(3,4)P_2_ synthesis at endocytic CCPs. **c**, Representative total internal reflection (TIRF) microscopy images of Cos7 cells stably expressing eGFP–clathrin light chain and treated with DMSO, PITCOIN1 or PITCOIN3 (20 µM, 6 h). Cells were stained for endogenous clathrin (CHC) and PI(3,4)P_2_. Scale bars, 50 µm. The insets show zoomed images. Colocalization of clathrin and PI(3,4)P_2_ is indicated by arrowheads. Scale bars, 10 µm. **d**, Quantification of PI(3,4)P_2_ levels at CCPs in fixed Cos7 cells treated with DMSO, PITCOIN1 or PITCOIN3 (20 μM, 6 h). Mean ± s.e.m.; *n* = 3; **P *<* 0.01 (DMSO versus PITCOIN1, *P* = 0.0022; DMSO versus PITCOIN3, *P* = 0.0020); one-way ANOVA with Tukey’s multiple-comparisons test. **e**, PITCOIN1 and PITCOIN3 impair clathrin-mediated endocytosis of transferrin. Ratio of internalized to surface transferrin in Cos7 cells treated with DMSO, PITCOIN1 or PITCOIN3 (20 µM, 6 h) or depleted of endogenous PI3KC2α by specific small interfering RNA (siRNA). Mean ± s.e.m.; *n* = 3; **P *<* 0.01, ***P *<* 0.001 (DMSO versus siRNA PI3KC2α, *P* = 0.0021; DMSO versus PITCOIN1, *P* = 0.0026; DMSO versus PITCOIN3, *P* = 0.001); one-way ANOVA with Tukey’s multiple-comparisons test. **f,g**, Inhibition of PI3KC2α stalls endocytic CCP dynamics. **f**, Representative kymographs of clathrin fluorescence in Cos7 cells stably expressing eGFP–clathrin light chain over 180 s monitored by TIRF imaging. Clathrin dynamics are reduced in cells treated with PITCOIN1 and PITCOIN3 (20 µM, 6 h). **g,** Time constants of clathrin-coated endocytic structures analyzed by quantitative automated two-dimensional (2D)-tracking. Data from individual cells are displayed as red circles. Mean ± s.e.m.; unpaired two tailed *t*-test; *P < 0.05 (*P* = 0.03) and **P < 0.01 (*P* = 0.004). Source data

**Extended Data Fig. 5:**
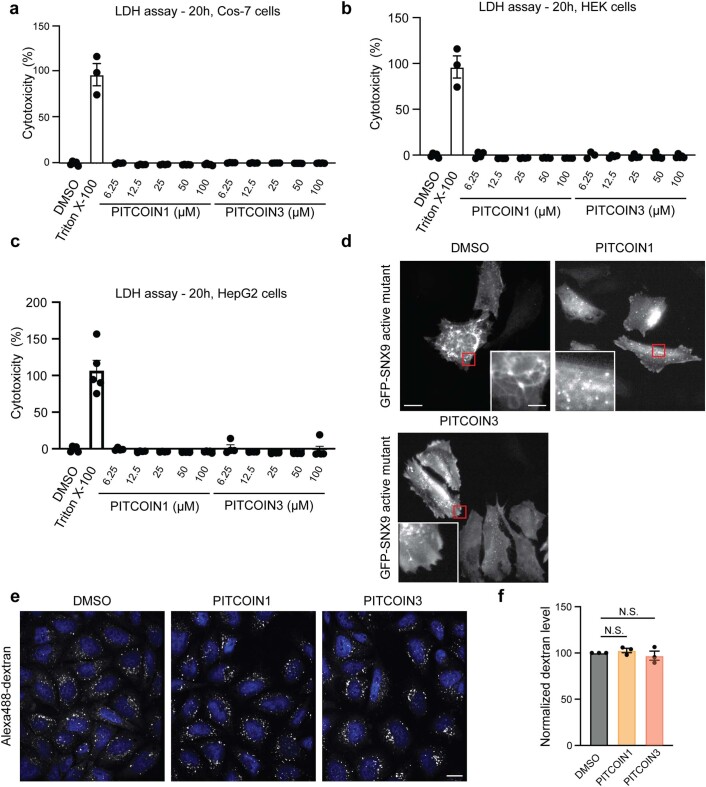
Small molecule inhibition of PI3KC2α impairs SNX9-mediated plasma membrane tubulation. (**a**–**c**) PITCOIN1 and PITCOIN3 are non-toxic to mammalian cells. Cos-7 (**a**), HEK (**b**), or HepG2 (**c**) cells were treated for 20 h with the indicated concentrations of PITCOIN1 or PITCOIN3 and subjected to lactate dehydrogenase (LDH) assays. Triton X-100 served as a positive control for cytotoxicity. n = 4 independent experiments. Data are presented as Means ± SEM. (**d**) PITCOIN1 and PITCOIN3 treatment of HeLa cells overexpressing hyperactive eGFP-SNX9. Representative images of DMSO-, PITCOIN1- or PITCOIN3-treated HeLa cells (20 μM for 6 h, n = 3 individual experiments) expressing hyperactive eGFP-SNX9. PITCOIN treatment abrogated formation of eGFP-SNX9-induced plasma membrane tubules. Insets show magnified views of boxed area. Scale bars, 10 μm or 2 μm (inset). (**e**, **f**) PITCOIN1 and PITCOIN3 do not affect fluid-phase endocytosis of dextrans. (**e**) Representative confocal microscopy images of HeLa cells treated with DMSO, PITCOIN1, or PITCOIN3 (20 μM for 6 h) and incubated with Alex488-conjugated dextran for 60 mins. Scale bar, 10 μm. (**f**) Quantification of representative data shown in (**e**). Data are from n = 3 independent experiments and depicted as mean ± SEM. N.S., non-significant, one-way ANOVA with Tukey’s multiple comparison test. Source data

To assess the use of PITCOINs for targeting PI3KC2α activity in mammalian cells, we determined whether these compounds might be cytotoxic. Sustained exposure for 20 h of HeLa cells to different concentrations of PITCOIN1 or PITCOIN3 up to 100 µM in the presence or absence of serum did not result in detectable cytotoxicity as measured by trypan blue staining (Fig. 3a). PITCOINs also were nontoxic if analyzed by the lactate dehydrogenase (LDH) assay in a variety of different cell types, for example, Cos7, HEK293 and HepG2 cells (Extended Data Fig. 5a–c).

**Extended Data Fig. 6:**
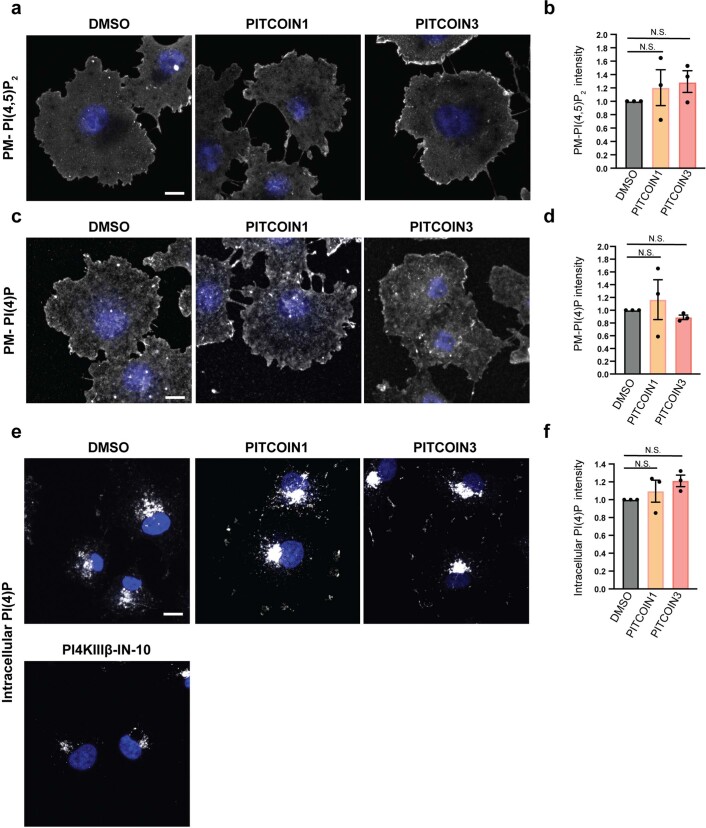
PITCOIN1 and PITCOIN3 do not affect cellular PI(4,5)P_2_ and PI(4)P levels. (**a**) Representative confocal microscopy images of fixed Cos7 cells treated with DMSO, PITCOIN1, or PITCOIN3 (20 μM for 6 h) and stained for PI(4,5)P_2_ using specific antibodies. (**b**) Quantification of representative data shown in (**a**). Data are from n = 3 independent experiments and depicted as mean ± SEM. N.S., non-significant, one-way ANOVA with Tukey’s multiple comparison test. (**c**) Representative confocal microscopy images of fixed Cos7 cells treated with DMSO, PITCOIN1, or PITCOIN3 (20 μM for 6 h) and stained for PI(4)P on the cell surface using specific antibodies. (**d**) Quantification of representative data shown in (**c**). Data are from n = 3 independent experiments and depicted as mean ± SEM. N.S., non-significant, one-way ANOVA with Tukey’s multiple comparison test. (**e**) Representative confocal microscopy images of fixed Cos7 cells treated with DMSO, PITCOIN1, or PITCOIN3 (20 μM for 6 h) and stained for internal pools of PI(4)P using specific antibodies. Cells treated with 5 µM PI4KIIIβ-IN-10 for 2 h were used as a positive control. (**f**) Quantification of representative data shown in (**e**). Data are from n = 3 independent experiments and depicted as mean ± SEM. N.S., non-significant, one-way ANOVA with Tukey’s multiple comparison test.

Previous studies have shown that PI3KC2α synthesizes a local plasma membrane pool of PI(3,4)P_2_ that drives endocytic membrane remodeling by the PI(3,4)P_2_-binding PX-BAR domain protein sorting nexin 9 (SNX9)^15,33,34^. Consistent with this, pharmacological inhibition of PI3KC2α activity in the presence of PITCOIN1 or PITCOIN3 led to a progressive dose-dependent block of plasma membrane tubule formation induced by constitutively active enhanced green fluorescent protein (eGFP)-SNX9 with half-maximal effective concentration (EC_50_) values of 5.0 and 4.6 µM, respectively (Fig. 3b and Extended Data Fig. 5d), that is, phenocopying genetic depletion of PI3KC2α in cells^33^. To probe the cellular effects of PITCOINs on PI3KC2α-mediated synthesis of PI(3,4)P_2_ directly, we monitored the local levels of PI(3,4)P_2_ at endocytic clathrin-coated pits (CCPs) in Cos7 cells expressing eGFP–clathrin light chain. Acute perturbation of PI3KC2α activity by pretreatment of cells with PITCOIN1 or PITCOIN3 depleted PI(3,4)P_2_ from endocytic CCPs (Fig. 3c,d) and resulted in impaired clathrin-mediated endocytosis of transferrin, akin to genetic loss of PI3KC2α (Fig. 3e). Clathrin-independent fluid-phase uptake of fluorescent large dextrans^35^ proceeded unperturbed in the presence of PITCOINs (Extended Data Fig. 5e,f). Depletion of PI(3,4)P_2_ from CCPs and inhibition of transferrin endocytosis were accompanied by attenuated dynamics of CCPs as evidenced by a significant increase in CCP lifetime (Fig. 3f,g), that is, a hallmark of PI3KC2α deficiency^15,16,36^. In contrast, synthesis of plasma membrane PI(4,5)P_2_ or PI(4)P was unaffected by PITCOIN1 or PITCOIN3 (Extended Data Fig. 6a–d). Application of PITCOINs also had no effect on the Golgi pool of PI(4)P, which is mainly synthesized by PI 4 kinase IIIβ^37^ (Extended Data Fig. 6e,f).

These results demonstrate that PITCOIN1 and PITCOIN3 potently and specifically inhibit the activity of PI3KC2α but no other PI kinases and, thereby, impair endocytic plasma membrane remodeling in living mammalian cells.

### PI3KC2α contributes to endosomal PI(3)P synthesis

**Fig. 4:**
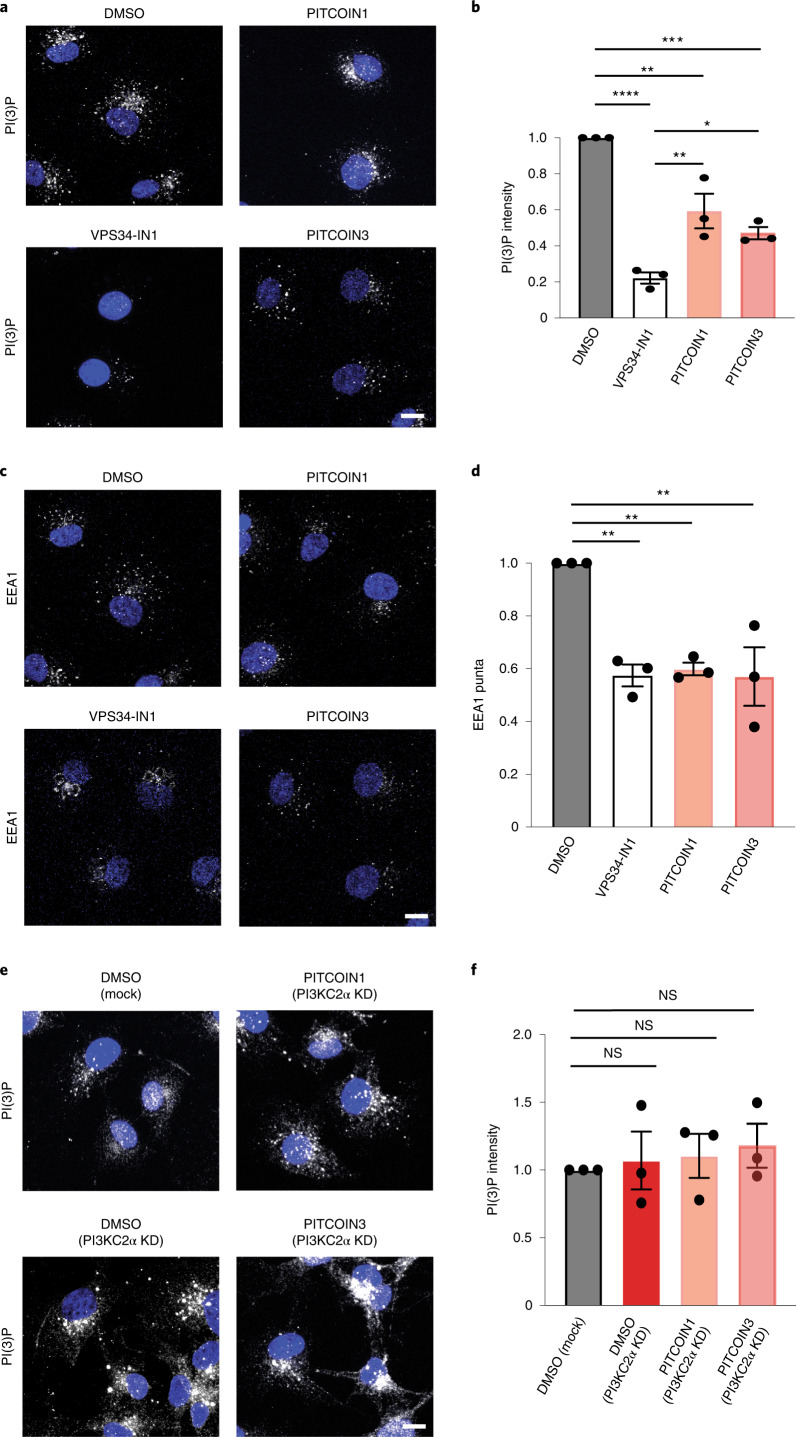
PI3KC2α contributes to endosomal PI(3)P synthesis. **a**,**b**, Pharmacological inhibition of PI3KC2α reduces endosomal PI(3)P levels in Cos7 cells. **a**, Representative confocal microscopy images of fixed Cos7 cells treated with DMSO, PITCOIN1, PITCOIN3 (20 µM for 6 h) or VPS34-IN1 (10 µM for 1 h) and stained for PI(3)P using recombinant eGFP–2×FYVE domain of Hrs. Scale bar, 10 µm. **b**, Quantification of representative data shown in **a**. Data are from *n* = 3 independent experiments. Mean ± s.e.m.; *P < 0.05, **P < 0.01, ***P < 0.0001, ****P < 0.0001 (DMSO versus PITCOIN1, *P* = 0.003; DMSO versus PITCOIN3, *P* = 0.005; VPS34-IN1 versus PITCOIN1, *P* = 0.0051; VPS34-IN1 versus PITCOIN3, *P* = 0.0444); one-way ANOVA with Tukey’s multiple-comparisons test. **c**,**d**, Pharmacological inhibition of PI3KC2α reduces EEA1 recruitment to endosomes. **c**, Representative confocal microscopy images of fixed Cos7 cells treated with DMSO, PITCOIN1, PITCOIN3 (20 µM for 6 h) or VPS34-IN1 (10 µM for 1 h) and immunostained for EEA1. Scale bar, 10 µm. **d**, Quantification of representative data shown in **c**. Data are from *n* = 3 independent experiments. Mean ± s.e.m.; **P < 0.01 (DMSO versus VPS34-IN1, *P* = 0.0048; DMSO versus PITCOIN1, *P* = 0.0068; DMSO versus PITCOIN3, *P* = 0.0045); one-way ANOVA with Tukey’s multiple-comparisons test. **e**,**f**, Endosomal PI(3)P levels are unaltered in PI3KC2α-depleted Cos7 cells treated for 6 h with PITCOIN1 or PITCOIN3. **e**, Representative confocal microscopy images of fixed Cos7 cells depleted of endogenous PI3KC2α using specific siRNA, treated with DMSO, PITCOIN1, PITCOIN3 (20 µM for 6 h) or VPS34-IN1 (10 µM for 1 h) and stained for PI(3)P using recombinant eGFP–2×FYVE domain of Hrs. Scale bar, 10 µm. KD, knockdown. **f** Quantification of representative data shown in **e**. Data are from *n* = 3 independent experiments. Mean ± s.e.m.; NS, not significant; one-way ANOVA with Tukey’s multiple-comparisons test. Source data

**Extended Data Fig. 7:**
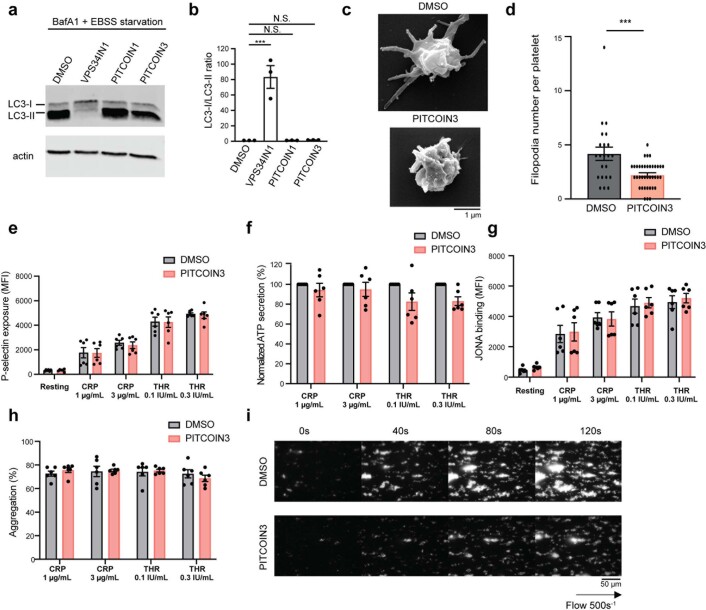
PITCOIN1 and PITCOIN3 do not affect PI3KC2α-independent functions. (**a**, **b**) PITCOIN1 and PITCOIN3 do not affect starvation-induced autophagosome formation. HEK293 cells were pretreated with DMSO (0.1%), VPS34-IN1 (5 μM, 2 hours), or PITCOIN1 and PITCOIN3 (20 μM, 5 hours). Autophagy was induced by starvation for 1 hour in EBSS in the continued presence of DMSO or the above inhibitors and bafilomycin A1 (100 nM). Cell lysates were then analyzed by SDS-PAGE and immunoblotting for LC3-I/II and β-actin as a loading control. (**b**) Quantification of representative immunoblotting data shown in (**a**) depicted as the ratio of LC3-I/LC3-II. Data are from n = 3 independent experiments and depicted as mean ± SEM. ***p < 0.001 (p = 0.0002), N.S., non-significant, one-way ANOVA with Tukey’s multiple comparison test. (**c**) Scanning electron microscopy analysis of mouse platelets incubated with DMSO or PITCOIN3 (20 µM, 6 h) followed by activation with CRP (1µg/ml for 3 minutes). Representative images are shown. Scale bar, 1 µm. (**d**) Filipodia number per platelets was quantified using ImageJ. Mean ± SEM from two independent experiments (n: DMSO = 22 platelets; PITCOIN3=38 platelets); ***p < 0.001 (p = 0.0005, two-tailed) vs DMSO, unpaired student t-test. (**e**–**h**) PITCOIN3 does not affect PI3KC2α-independent platelet functions. Washed platelets were treated with vehicle (DMSO) or 20 µM PITCOIN for 6 hours and stimulated with the indicated doses of CRP or thrombin (THR). PITCOIN3 treatment does not affect P-selectin exposure to the plasma membrane (**e**), ATP release (**f**), αIIbβ3-integrin activation by JONA binding (**g**), or platelet aggregation (**h**). Data represent mean ± S.E.M, n = 6 mice, two-way ANOVA test followed by a Sidak’s multiple comparisons. (**i**) Thrombus formation. Mouse whole blood samples from n = 4 mice per condition, in which platelets were labeled with DIOC_6_, were incubated with DMSO or PITCOIN3 (20 µM, 6 hours) and perfused through a collagen-coated microcapillary at an arterial shear rate of 500 s^−1^ during 2 minutes. Images are representative of thrombi formation in real time. Scale bar, 50 μm. Source data

Apart from the synthesis of PI(3,4)P_2_ at plasma membrane endocytic sites and at the cytokinetic bridge^20^, PI3KC2α has been implicated in contributing to the formation of endosomal pools of PI(3)P in a cell-type- and/or context-specific manner^7,12,14,17,21,27,38^. A major alternative route of PI(3)P formation on endosomes involves Vps34 (refs. ^4,37^), an enzyme that can be selectively inhibited by VPS34-IN1 (ref. ^9^). To assess the contribution of PI3KC2α and Vps34 to the overall synthesis of PI(3)P on endosomes, we monitored the cellular levels of PI(3)P in Cos7 cells treated with PITCOINs or VPS34-IN1 using the recombinant eGFP-2×FYVE domain of Hrs as a sensor. Inhibition of Vps34 in the presence of VPS34-IN1 greatly reduced endosomal PI(3)P levels, consistent with prior data^8,9,39^. Interestingly, pharmacological blockade of PI3KC2α by either PITCOIN1 or PITCOIN3 also led to a significant, albeit less pronounced, reduction in endosomal PI(3)P (Fig. 4a,b). Endosomal membrane recruitment of the PI(3)P-binding effector early endosomal antigen 1 (EEA1) was concomitantly reduced (Fig. 4c,d). This was surprising as previous work by us had failed to detect significant changes in PI(3)P levels in Cos7 cells with sustained genetic depletion of PI3KC2α^16^. To rule out possible off-target effects of PITCOINs on other pathways of PI(3)P synthesis, we tested the effect of PITCOINs on endosomal PI(3)P in PI3KC2α-depleted Cos7 cells (Fig. 4e,f). Lack of PI3KC2α had no effect on PI(3)P, corroborating our earlier data^16^. Notably, application of PITCOIN1 or PITCOIN3 failed to elicit changes in PI(3)P levels in PI3KC2α-depleted Cos7 cells, confirming their exquisite PI3KC2α target specificity. Furthermore, treatment of starved Cos7 cells with PITCOIN1 or PITCOIN3 did not affect the formation of LC3-II-containing autophagosomes, a process that largely depends on Vps34-mediated formation of an autophagy-specific pool of PI(3)P in most cell types (Extended Data Fig. 7a,b).

These results show that PI3KC2α contributes to endosomal PI(3)P synthesis, either directly by phosphorylating PI or indirectly via production of PI(3,4)P_2_, which can subsequently be hydrolyzed to PI(3)P by endosomal INPP4A/INPP4B 4-phosphatases^3,16^. They further suggest that sustained genetic loss of PI3KC2α in Cos7 fibroblasts leads to compensatory changes in PI(3)P metabolism, for example, via regulation of lipid phosphatases that remain to be identified.

### PITCOIN3 impairs platelet membrane and thrombus formation

A major cell type reported to depend on PI(3)P synthesis mediated by PI3KC2α is platelets^27–29^, megakaryocyte-derived blood cells of major biomedical relevance due to their physiological role in thrombus formation and, thus, as a target for antithrombotic drugs. Previous studies in kinase-inactive PI3KC2α knock-in mice have revealed a critical role for the enzyme in controlling platelet membrane morphology and function via synthesis of an agonist-insensitive pool of PI(3)P^27,29^. Moreover, application of PIK-90-derived PI3K inhibitors with activity against PI3KC2α and other PI3Ks including PI3KC2β and class I PI3Kα has provided proof of principle that acute perturbation of PI3KC2α function may impair thrombosis without suppression of canonical platelet activation mechanisms that affect bleeding^28^. The interpretation of these data is, however, compromised by the off-target activities of the broad-spectrum PI3K inhibitors used.

**Fig. 5:**
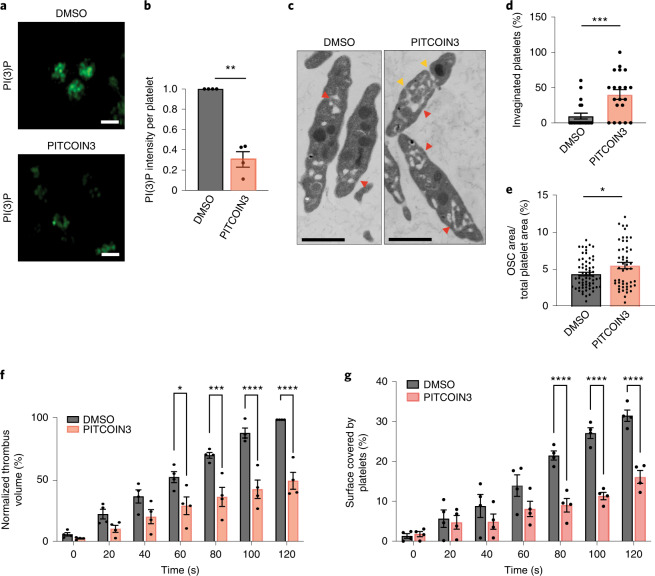
PI3KC2α inhibition by PITCOINs impairs platelet membrane remodeling and thrombus formation. **a**, Representative confocal images of PI(3)P levels in mouse resting platelets treated with DMSO or PITCOIN3 (20 µM, 6 h). Platelets were fixed and stained with PI(3)P-specific antibody followed by Alexa488-conjugated secondary anti-mouse antibody for detection. Scale bars, 2 µm. **b**, Quantification of representative data shown in **a** using ImageJ. Mean ± s.e.m.; data are from *n* *=* 4 independent experiments; **P < 0.01 (*P* = 0.0029); two-tailed, one-sample *t*-test with hypothetical mean value of 1.0. **c**, Transmission EM analysis of mouse resting platelets incubated with DMSO or PITCOIN3 (20 µM, 6 h). Representative images are shown. Scale bars, 1 µm. Red arrowheads, OCS; orange arrowheads, invaginated membranes. **d**,**e**, Quantification of representative data shown in **c**. **d**, Fraction of invaginated platelets quantified using ImageJ. Mean ± s.e.m.; *n* *=* 5 fields from each of four independent experiments; ***P < 0.001 (*P* = 0.0005) versus DMSO, two-tailed unpaired Student’s *t*-test. **e**, OCS area as a fraction of total platelet area. Mean ± s.e.m.; *n* = 4 independent experiments (DMSO, 63 platelets; PITCOIN3, 50 platelets); *P < 0.05 (*P* = 0.0183) versus DMSO, two-tailed unpaired Student’s *t*-test. **f**, Thrombus volume was measured using Imaris. Mean ± s.e.m.; *n* *=* 4 mice per condition. *P < 0.05 (*P* = 0.0133), ***P < 0.001 (*P* = 0.001) and ****P < 0.0001 versus DMSO, two-way ANOVA followed by Sidak’s multiple comparisons. **g**, Surface covered by platelets measured using ImageJ. Mean ± s.e.m.; *n* = 4 mice per condition. ****P < 0.0001 versus vehicle, two-way ANOVA followed by Sidak’s multiple comparisons test. Source data

Based on these prior works, we hypothesized that acute specific inhibition of PI3KC2α activity by PITCOIN3 should recapitulate key phenotypes elicited by genetic PI3KC2α kinase inactivation and counteract thrombosis. Consistent with this hypothesis, we observed a dramatic decrease in basal PI(3)P levels in platelets treated with PITCOIN3 (Fig. 5a,b), illustrating the important role of PI3KC2α in PI(3)P production in platelets. Ultrastructural analysis of PITCOIN3-treated platelets by transmission electron microscopy (EM) revealed major defects in membrane morphology, including an aberrant invaginated shape of the plasma membrane (Fig. 5c,d). The open canalicular system (OCS) that constitutes a reservoir of plasma membrane to enable platelet shape changes during activation was also altered and expanded (Fig. 5e). Moreover, acute inhibition of PI3KC2α activity by PITCOIN3 caused dramatic defects in filopodia extension in collagen-related peptide (CRP)-stimulated platelets visualized by scanning EM (Extended Data Fig. 7c,d). Platelet processes known to be independent of PI3KC2α^27^ such as CRP- or thrombin-stimulated P-selectin exposure (Extended Data Fig. 7e), ATP release (Extended Data Fig. 7f), αIIbβ3-integrin activation (Extended Data Fig. 7g) or platelet aggregation (Extended Data Fig. 7h) were not affected significantly by PITCOIN3. Hence, acute inhibition of PI3KC2α by PITCOIN3 selectively causes major defects in platelet membrane morphology, thereby recapitulating key features of genetically induced PI3KC2α activity loss in mice^27,29^.

We therefore tested whether PITCOIN3 displays antithrombotic activity in mouse blood. To this end, we analyzed platelet-dependent thrombus formation ex vivo on a collagen matrix at an arterial shear rate of 500 s^−1^ during 2 min of perfusion. We found that acute inhibition of PI3KC2α activity in the presence of PITCOIN3 caused a pronounced and highly significant reduction in the prothrombotic capacity of platelets compared to DMSO-treated controls (Fig. 5f and Extended Data Fig. 7i). Moreover, the surface covered by platelets was significantly decreased (Fig. 5g).

These data corroborate the key physiological role of PI3KC2α in controlling platelet membrane morphology and function and identify PITCOIN3 as a lead compound for further development as a potential antithrombotic drug.

## Discussion

We have combined HTS with subsequent medicinal chemistry approaches to identify potent and selective small-molecule inhibitors of PI3KC2α activity and provide proof of principle for their use in dissecting the multiple roles of this enzyme in cell physiology and to combat human disease.

Multiple independent lines of evidence support the notion that PITCOIN1–PITCOIN3 potently and specifically inhibit the activity of PI3KC2α but no other PI kinases in vitro and in living cells. PITCOINs were found to be inactive against other PI3Ks including PI3Kα and Vps34 in vitro, and further selectivity profiling revealed no off-target activity of PITCOIN1–PITCOIN3 toward any of more than 117 lipid and protein kinases studied. Our MS-based Kinobead profiling showed selective competition of PIK3C2α among the 137 quantified kinases in HEK293T cells. Structural studies by protein X-ray crystallography demonstrate that PITCOIN molecules display a unique mode of interaction with the ATP-binding site of PI3KC2α. Complex formation is found to involve key interactions between the R^1^ and R^2^ arms of PITCOIN molecules and target residues that are not conserved in other class II PI3K isoforms (Fig. 2), providing a molecular explanation for the potency and unprecedented high selectivity of these molecules for PI3KC2α. We note that this binding mode is distinct from the off-target association of pan-PIK inhibitors such as PIK-90 with PI3KC2α, which capitalizes exclusively on binding interfaces that are conserved between PI3K family members^30^. Notably, we demonstrate that the cellular application of PITCOIN1 or PITCOIN3 recapitulates key phenotypes elicited by genetic loss of PI3KC2α ^7,14,15,17,21,29^. Specifically, we show that acute pharmacological inhibition of PI3KC2α activity causes the local depletion of PI(3,4)P_2_ from CCPs, resulting in impaired endocytic plasma membrane remodeling, and reduces endosomal PI(3)P levels (Figs. 3 and 4). These phenotypes are abrogated in the absence of the target enzyme. Finally, experiments in platelets show that acute pharmacological targeting of PI3KC2α recapitulates key features of genetically induced PI3KC2α activity loss in mice^27,29^, that is, defects in platelet membrane morphology caused by depletion of PI(3)P and a prominent reduction in prothrombotic capacity (Fig. 5).

PITCOINs thus are first-in-class potent and selective inhibitors of PI3KC2α catalytic function and represent unique pharmacological tools to further decipher the multiple roles of PI3KC2α in biology. The observation that acute inhibition of PI3KC2α causes antithrombotic effects that occur concomitantly with a reduction in PI(3)P levels and changes in platelet membrane morphology (that is, the OCS) opens the possibility that PITCOIN3 and related compounds may serve as a new class of shear stress-dependent^12,27^ antithrombotic agents that, unlike current therapies, may not increase bleeding risk. Future studies will be directed to explore this exciting perspective and to further improve PITCOINs, for example, with respect to cell membrane permeability. In addition, PITCOINs and their derivatives could also provide new therapeutic avenues for the treatment of other important human diseases related to PI3KC2α function, including viral infection^25,26^, diabetes^21^ or cancer^1,7^ that pertain to the established physiological roles of PI3KC2α in viral replication^25,26^, VEGF signaling and endocytosis^15,17^ and the abscission reaction of cytokinesis^23^, respectively.

Finally, the structure-based development of selective PI3KC2α inhibitors reported here will serve as a door opener for the pharmacological targeting of other class II PI3Ks (that is, PI3KC2β and PI3KC2γ) with key roles in mTORC1 signaling^40^, cell migration^38,41^, inherited muscle disease, stroke^42^ and insulin signaling or metabolism^43^.

## Methods

### Animals

All mice were C57BL/6J background males purchased from Janvier Labs. Mice were housed in conventional cages under specific pathogen-free conditions in an animal room with constant temperature (20–22 °C) and humidity (50–60%) with a 12 h light/12 h dark cycle (lights on at 7:00 AM) and free access to food and water. All procedures were performed in accordance with institutional guidelines for animal research and were approved by the French Ministry of Research in agreement with European Union guidelines.

### Preparation of mouse-washed platelets

Whole blood was drawn from the inferior vena cava of mice anesthetized with a mixture of Imalgene (25 mg kg^−1^) (Merial) and Rompun (10 mg kg^−1^) (Bayer) into a syringe containing acid citrate dextrose (3% trisodium citrate × 5.5H_2_0, 1.4% citric acid, 2% glucose) (1 anticoagulant volume per 9 volumes of blood). Platelet-rich plasma (PRP) was obtained by mixing blood with 1 volume of modified HEPES-Tyrode’s buffer (140 mM NaCl, 2 mM KCl, 12 mM NaHCO_3_, 0.3 mM NaH_2_PO_4_, 1 M MgCl_2_, 5.5 mM glucose, 5 mM HEPES; pH 6.7) containing 0.35% BSA followed by centrifugation at 300*g* for 4 min. After PGI_2_ (Sigma-Aldrich) addition at a final concentration of 500 nM to the PRP, platelets were pelleted by centrifugation at 1,000*g* for 6 min, resuspended in modified HEPES-Tyrode’s buffer (pH 7.38) in the presence of 0.02 IU ml^−1^ of the ADP scavenger apyrase (Sigma-Aldrich) and rested for 45 min at 37 °C.

### Oligonucleotides

Oligonucleotide sequences used in this study are listed in Supplementary Table 4.

### Antibodies

The following antibodies were used in this study (dilution given in parentheses):

#### Primary antibodies used for immunocytochemistry

Mouse PI(3,4)P_2_ IgG (Echelon Biosciences, 1:600), mouse PI(4,5)P_2_ IgM (Echelon Biosciences, 1:400), mouse PI(4)P IgM (Echelon Biosciences, 1:70), mouse PI(3)P IgG (Echelon Biosciences, 1:100), rabbit anti-EEA1 (Cell Signaling, 1:100), LC3B antibody (Novus NB100-2220, 1:1,000), mouse anti-GFP (Clontech, 1:400) and rat PE-conjugated JON/A antibody (Emfret Analytics M023-2, 1:5)

#### Secondary antibodies

Goat anti-mouse IgG (H+L) AF488 (ThermoFisher, 1:400), goat anti-rabbit IgG (H+L) AF647 (ThermoFisher, 1:400), goat anti-mouse IgM AF568 (ThermoFisher, 1:400) and IRDye 800CW goat anti-rabbit IgG (LI-COR, 1:5,000)

### Cell lines

HeLa (CCL-2), HEK293T (CRL-11268) and Cos7 (CRL-1651) cells were obtained from ATCC and cultured in DMEM with 4.5 g l^−1^ glucose (Lonza) containing 10% heat-inactivated FBS, 100 U ml^−1^ penicillin and 100 μg ml^−1^ streptomycin (Gibco). Cells were routinely tested for and devoid of mycoplasma contamination.

### Cloning and mutagenesis

Constructs for baculovirus-mediated expression in insect cells, cDNA encoding human PI3KC2α^ΔN^ (amino acids 376–1686) amplified by PCR and cloned into pFL10His via KasI/XbaI restriction sites and mouse PI3KC2α^core^ (amino acids 377–1400; engineered internal loop with amino acids 533–544 replaced by GSGS; HBD (amino acids 550–665) replaced by the sequence SGAGSGA) were engineered by multiple steps of site-directed mutagenesis using PCR.

### Protein expression and purification

His_10_-tagged PI3KC2α^ΔN^ and PI3KC2α^core^ were expressed in *Sf*21 insect cells, using SF900-II serum-free medium (ThermoFisher). *Sf*21 cells (800 ml) grown to a density of 1.5–2 × 10^6^ cells per ml were infected with 8 ml amplified baculovirus encoding the desired construct. Cells were collected around 72 h after virus infections. The cell viability was around 80–90%. Cell pellets were stored frozen at −20 °C until purification. For protein purification, cell pellets from each 200-ml culture were resuspended in 35 ml lysis buffer (50 mM Tris pH 7.2, 300 mM NaCl, 10 mM imidazole, 1 mM DTT, 0.5% Triton X-100, 1 tablet per 50 ml of protease inhibitor cocktail), sonicated for 1 min (1-s pulse on, 5-s pulse off) and centrifuged for 20 min at 87,200*g*. Each 50 ml of supernatant was incubated with 0.5 ml nickel NTA beads (Sigma) on a rotating wheel for 1 h at 4 °C. Beads were collected in a plastic open column (Bio-Rad) and washed with 20 ml lysis buffer and then with 30 ml wash buffer (50 mM Tris pH 7.5, 300 mM NaCl, 20 mM imidazole, 1 mM DTT). Protein was eluted with 8 ml elution buffer (20 mM Tris pH 7.5, 300 mM NaCl, 300 mM imidazole, 5 mM DTT). The His_10_ tag was released by Tobacco Etch Virus nuclear-inclusion-a endopeptidase (TEV) (10 mg protein per 0.25 mg TEV) cleavage overnight, while dialyzing against size exclusion chromatography (SEC) buffer (20 mM Tris pH 7.5, 300 mM NaCl, 5 mM DTT) at 4 °C. Proteins were purified on a Superdex 200 gel filtration column at 4 °C with SEC buffer (20 mM Tris pH 7.5, 300 mM NaCl, 5 mM DTT). Proteins were concentrated to about 1.2 mg ml^−1^ for human PI3KC2α^ΔN^ and 5 mg ml^−1^ for mouse PI3KC2α^core^. Purified human PI3KC2α^ΔN^ was flash frozen in liquid nitrogen and stored at −80 °C, and mouse PI3KC2α^core^ was immediately used for crystallization. VPS34 complex II was purified as described previously^44^.

### Compound library screening and IC_50_ determination

Primary screens were performed using the ADP-Glo assay in a 384-well format. In total, 37,224 small molecules from the FMP library were screened for their ability to inhibit the lipid kinase activity of purified PI3KC2α. The concentration of each compound used in the primary screens was 10 μM. Kinase incubations with ATP but without PI were used as negative control samples. For IC_50_ determination, purified human PI3KC2α^ΔN^ was prediluted to 0.5 mg ml^−1^ in SEC buffer used for purification and subsequently diluted to 30 μg ml^−1^ in kinase buffer (5 mM HEPES/KOH pH 7.2, 25 mM KCl, 2.5 mM magnesium acetate, 150 mM potassium glutamate, 10 μM CaCl_2_, 0.2% CHAPS). Native liver PI was dissolved to a concentration of 1,000 µM with kinase buffer by water bath sonification and then suppliment with 50 μM ATP for IC_50_ measurement. A 2× compound dilution series (with a total of 11 concentrations) starting from 10 μM was prepared in kinase buffer. Reactions were started by mixing 5 μl of compound with 3 μl of purified PI3KC2α^ΔN^ for 5 min, followed by initiation of kinase reactions by adding 2 μl of substrates. The kinase reactions were performed for 20 min at room temperature. The reactions were stopped by adding 10 μl of ADP-Glo reagent (Promega). After incubation for 40 min, 20 μl of kinase detection reagent was added. After a further incubation for 20 min, luminescence was read with a TECAN plate reader. IC_50_ was calculated according to the manufacturer’s protocol for the ADP-Glo assay.

### Chemical synthesis of PITCOINs

Chemical synthesis of PTCOINs was performed as indicated in the Supplementary Note.

### Kinobead profiling

Kinobead pulldown assays were performed as previously described using 5 mg ml^−1^ HEK293 cell lysates in IGEPAL CA-630-containing buffer^32^. Briefly, for profiling of each PITCOIN, 12 wells of a 96-well plate were filled with lysate (2.5 mg of total proteins per well) and incubated for 45 min at 4 °C in an end-over-end shaker with 0 nM (DMSO control), 30 nM, 100 nM, 300 nM, 1 μM, 3 μM, 10 μM, 30 μM, 100 μM, 300 μM and 1 mM of the PITCOINs dissolved in DMSO. Subsequently, the treated lysates were incubated with Kinobeads-ε for 30 min at 4 °C in a 96-well filter plate on an end-over-end shaker. The beads were then washed before the bound proteins were denatured and alkylated with chloroacetamide. Addition of trypsin (300 ng per well) started overnight on-bead digestion. Acidified peptide eluates were then subjected to C18 StageTip desalting^45^ for LC–MS/MS analysis on an Orbitrap Fusion Lumos Tribrid (ThermoFisher Scientific) mass spectrometer coupled to an online Dionex Ultimate3000 equipped with a micro flow Vanquish pump UHPLC (ThermoFisher Scientific). MaxQuant (v.1.5.3.30.)/Andromeda was used to quantify proteins using the Swissprot reference database containing all canonical protein sequences with standard settings. Dose–response curves, Kdapps and the kinome tree were derived from the proteinGroup.txt file using a set of R scripts^46^. The MS proteomics data have been deposited to the ProteomeXchange Consortium (http://proteomecentral.proteomexchange.org) via the PRIDE^47^ partner repository dataset identifier PXD032284.

### Crystallization of PI3KC2α^core^

Diffracting crystals of mouse PI3KC2α^core^ were obtained as described previously ^30^. In brief, concentrated protein samples (2.5 mg ml^−1^) were complexed with 0.5 mM of PITCOIN1, PITCOIN2 or PITCOIN3 overnight on ice. Samples were filtered with 0.2-μm spin filters to remove precipitates. Crystals were grown in mother liquid containing 0.1 M Tris pH 7.5, 100–200 mM MgSO_4_ and 7–10% PEG 3,350. Crystals were washed and cryo-protected with mother liquid supplement with 10% ethylene glycol. Crystals were mounted in a nylon loop and flash cooled in liquid nitrogen.

### Data collection, model building and refinement

Diffraction data were collected at station BL14.1 of BESSY/Helmholtz Center Berlin (HZB). Images were processed with XDSAPP^48^. The CC_1/2_ cutoff of diffraction data was 0.996 (0.272), 0.998 (0.172) and 0.998 (0.251) with respect to the crystal of PI3KC2α ^core^ in complex with PITCOIN1, PITCOIN2 and PITCOIN3, respectively. Values in parentheses are for the highest-resolution shell. The PI3KC2α^ΔN+ΔC−C2^ structure was determined by molecular replacement with the PHENIX suite^49^ using the previously solved structure of PI3KC2α (PDB: 7BI4) as a search model. The structure was manually built using COOT and iteratively refined using Refmac^50^ and the PHENIX suite. The different orientations of the thiazole group in PITCOIN1, PITCOIN2 and PITCOIN3 were validated via a model fitting into the electron density map and further refinement of the model. Data collection and structure refinement statistics are summarized in Supplementary Table 2. The Ramachandran statistics in the order of favored, allowed and outliers for each structure are as follows: PI3KC2α ^core^ in complex with PITCOIN1 (97.15, 2.85, 0%), in complex with PITCOIN2 (96.80, 3.08, 0.12%) and in complex with PITCOIN3 (98.27, 1.73, 0%). The rotamer outlier percentage for each structure was as follows: PI3KC2α ^core^ in complex with PITCOIN1 (4.81%), in complex with PITCOIN2 (0%) and in complex with PITCOIN3 (0%).

### LDH cell toxicity assay

Toxicity was assayed by measurement of LDH released from cells due to cell death. Cos7 cells were seeded in a 96-well plate. The cells were treated with a concentration series of PITCOIN1 and PITCOIN3 for 20 h. The supernatant was collected and added to the LDH assay reagent (Sigma-Aldrich). The reactions were performed for 20 min. Absorbance was measured at 490 nm. Values were normalized to the drug/medium background value and converted into the percentage of toxicity with 100% lysed cell treated with Triton X-100 as a control.

### Analysis of CCP dynamics

A stable Cos7 cell line expressing eGFP–clathrin light chain was used for live cell imaging of CCP dynamics via TIRF microscopy. Time-lapse series of Cos7 cells pretreated with 20 µM compound for 6 h were recorded at a frame rate of 0.5 Hz for 3 min. Cells treated with 0.1% DMSO were used as a control. To quantitatively analyze CCP dynamics we used the associated cmeAnalysis MATLAB package^51^. Here the main features of the cmeAnalysis MATLAB package were combined to customized adaptations and newly written analysis functions in ref. ^52^. The analysis includes first detecting CCPs independently in individual frames and then tracking and closing tight gaps in between them using the functionality from the cmeAnalysis package. To ensure that trajectories of long-lived, low-motility structures with fluctuating intensities were not broken up into small segments, which would affect the result, the tracking parameters were adapted to the experimental data. As the dynamics of CCPs should be studied in this experiment, the CCP selection criteria were adjusted in a way that allowed analysis of true internalizations only. For this purpose, the large pool of short-lived (and often highly motile) structures of unclear origin were excluded. The same intention was kept for the subset of extremely long-lived CCPs, meaning that only structures with a duration above 20 s and below 160 s were included in lifetime analysis.

### Transferrin uptake and surface labeling

Cos7 cells were grown on Matrigel-coated glass coverslips and treated with 0.1% DMSO or 20 μM PITCOIN1 and PITCOIN3 for 6 h followed by starvation in serum-free DMEM for 1 h. For transferrin uptake, cells were incubated with 25 μg ml^−1^ Alexa647-labeled transferrin (Molecular Probes, Invitrogen) for 10 min at 37 °C in a humidity chamber. Cells were washed twice with ice-cold PBS supplemented with 10 mM MgCl_2_ and then washed with acid twice at pH 5.3 (0.2 M sodium acetate, 200 mM sodium chloride) on ice for 2 min to remove surface-bound transferrin. Cells were then washed another two times with ice-cold PBS containing 10 mM MgCl_2_ and fixed with 4% paraformaldehyde (PFA) for 45 min at room temperature. For surface labeling, cells were incubated with 25 mg ml^−1^ Alexa647-labeled transferrin at 4 °C for 45 min and then washed three times with ice-cold PBS (10 mM MgCl_2_) on ice for 1 min. Cells were fixed with 4% PFA for 45 min at room temperature. Transferrin labeling was analyzed using a Nikon Eclipse Ti microscope and ImageJ software. Internalized transferrin per cell was quantified and normalized to the amount of surface-bound transferrin determined in the same experiment as a measure for the efficiency of internalization.

### Fluid-phase endocytosis of fluorescent dextran

HeLa cells were grown on Matrigel-coated glass coverslips to near confluency before treatment for 6 h with 0.1% DMSO or 20 μM PITCOIN1 or PITCOIN3. Cells were incubated with 0.5 mg ml^−1^ Alexa488-labeled dextran (10k) (Thermo, 22910) in the continued presence of DMSO or PITCOINs for 60 min at 37 °C in a humidity chamber. Coverslips were washed three times with PBS for 2 min each before fixation for 45 min in 4% PFA. Cell nuclei were stained with Hoechst33342 (Thermo). Internalized dextran was quantitatively analyzed using a Nikon Eclipse Ti microscope equipped with ImageJ software.

### PI immunocytochemistry and imaging

#### PI(3)P detection in Cos7 cells

Cos7 cells were grown on Matrigel-coated glass coverslips and treated with 0.1% DMSO or 20 μM PITCOIN1 or PITCOIN3 for 6 h. Cells were washed with PBS containing 10 mM MgCl_2_ once and fixed in 2% PFA with 2% sucrose in PBS for 15 min at room temperature. Cells were washed three times with PBS containing 50 mM NH_4_Cl and permeabilized with PBS containing 20 μM digitonin (5% stock; Invitrogen) in buffer A (20 mM PIPES, pH 6.8, 137 mM NaCl, 2.7 mM KCl) for 5 min. Following three washes with buffer A, cells were stained for PI(3)P by incubation with the purified recombinant eGFP–2×FYVE domain of Hrs (0.5 μg ml^−1^) for 1 h at room temperature. Samples were stained with antibodies against GFP followed by labeling with Alexa488-conjugated secondary antibodies. Cells were imaged by laser scanning confocal microscopy, and PI(3)P levels were quantified using ImageJ software.

#### PI(3)P detection in platelets

Washed resting mouse platelets were adhered on poly(l-lysine)-coated slices for 30 min at 37 °C, fixed in 2% formaldehyde for 30 min and permeabilized with PIPES buffer (20 mM PIPES pH 6.8, 137 mM NaCl, 2.7 mM KCl) containing 20 µM digitonin for 5 min at room temperature. Following washes in PIPES buffer, samples were saturated with 10% goat serum in PIPES buffer for 1 h at room temperature and incubated with the anti-PI3P antibody (Echelon, 1:100) in 10% goat serum in PIPES buffer overnight at 4 °C followed by Alexa Fluor 488 anti-mouse antibody (Life Technologies, 1:500) in 10% goat serum in PIPES buffer for 1 h at room temperature. After several washes and slice mounting, samples were examined using the Zeiss LSM780 confocal microscope with a ×63, 1.4-NA Plan-Apochromat lens. PI(3)P levels were quantified using ImageJ software.

#### PI(3,4)P_2_ detection

Cos7 cells stably expressing eGFP–clathrin light chain were grown on Matrigel-coated glass coverslips and treated with 0.1% DMSO or 20 µM PITCOIN1 or PITCOIN3 for 6 h. Cells were washed with PBS containing 10 mM MgCl_2_ once and fixed in 2% PFA with 2% sucrose in PBS for 15 min at room temperature. Cells were washed three times with PBS containing 50 mM NH_4_Cl and permeabilized with PBS containing 0.5% saponin and 1% BSA for 30 min. Samples were incubated with primary (2 h) and secondary (1 h) antibodies diluted in PBS containing 1% BSA and 10% normal goat serum. Cells were analyzed by TIRF microscopy (Nikon TI Eclipse, 488- and 561-nm lasers, ×60 1.49-NA objective equipped with an sCMOS Andor mNeo camera). PI(3,4)P_2_ levels at CCPs were quantified using ImageJ software with eGFP–clathrin as a mask.

#### PI(4)P and PI(4,5)P_2_ detection

Cos7 cells were grown in matrix gel-coated glass coverslips and treated with 0.1% DMSO or 20 µM PITCOIN1 or PITCOIN3 for 6 h. Cos7 cells were washed with PBS containing 10 mM MgCl_2_ once. For detection of plasma membrane PI(4)P or PI(4,5)P_2_, cells were fixed in 2% PFA, 1% glutaraldehyde and 2% sucrose in PBS for 20 min at room temperature and permeabilized with PBS containing 0.5% saponin and 1% BSA in PBS for 30 min. For detection of the intracellular Golgi/TGN pool of PI(4)P, cells were fixed in 2% PFA and 2% sucrose in PBS for 20 min and permeabilized with 20 µM digitonin in buffer A for 5 min.

PI(4)P or PI(4,5)P_2_ was labeled using specific antibodies followed by detection with Alexa568-conjugated secondary antibodies. Cells were imaged by laser scanning confocal microscopy (Zeiss, LSM780). PI(4)P and PI(4,5)P_2_ levels were quantified using ImageJ software.

### Analysis of starvation-induced autophagosome formation

HEK293T cells were seeded at a density of 0.4 × 10^6^ cells per well into six-well plates coated with poly(l-lysine). Cells were grown overnight and pretreated with DMSO (0.1%, 6 h), VPS34-IN1 (5 µM, 2 h) or PITCOIN1 or PITCOIN3 (20 µM, 5 h) in DMEM containing 10% FBS. Cells were washed twice with EBSS, followed by co-treatment with bafilomycin A1 (100 nM) in EBSS for 1 h to block acidification. Cells were lysed in 200 μl TBS supplemented with 1% NP-40, 0.1% SDS and a protease inhibitor cocktail (Roche). Total protein concentrations were measured using the Bradford assay. Cell lysates (50 μg total protein) were analyzed by 15% SDS–PAGE and immunoblotting using antibodies against LC3-I, LC3-II and β-actin. IRDye 800CW-conjugated secondary antibodies were used for detection with a LI-COR imager system.

### EM analysis of platelet ultrastructure

#### Transmission EM

Washed mouse resting platelets were fixed in 2.5% glutaraldehyde in 0.05 M sodium cacodylate and 30 mM glucose at 4 °C for 24 h. Samples were embedded in 2% agarose, cut in 1-mm pieces, washed in 0.2 M sodium cacodylate buffer, postfixed with 1% osmium tetroxide in 0.2 M sodium cacodylate buffer for 1 h at room temperature and dehydrated in a series of graded ethanol solutions (30%, 50% and 70% 10 min each at room temperature) before being embedded in Embed 812 resin (Electron Microscopy Sciences) using a Leica EM AMW automated microwave tissue processor for EM. Samples were then sliced into 70-nm thick sections (Ultracut Reichert Jung) and mounted on 100-mesh copper grids before staining with 3% uranyl acetate in 50% ethanol and Reynold’s lead citrate. Examinations were carried out on a Hitachi HT7700 transmission electron microscope at an accelerating voltage of 80 kV. The OCS area and total platelet area were quantified using ImageJ software.

#### Scanning EM

Washed platelets were stimulated for 3 min with CRP and were fixed in 2.5% glutaraldehyde in 0.05 M sodium cacodylate and 30 mM glucose at 4 °C for 24 h. After resuspending in distilled water, platelets were allowed to adhere on poly(lysine)-coated coverglasses, rinsed and dehydrated in a graded ethanol series. Once dehydrated in 100% ethanol, samples were submitted to critical point drying with a Leica EM CPD 300. Finally, samples were coated with 6 nm platinium on a Leica EM Med 020 before being examined on an FEI Quanta 250 FEG scanning electron microscope at an accelerating voltage of 5 kV.

### Platelet flow assay on collagen

Microcapillaries were coated with Horm collagen suspension (100 μg ml^−1^) for 1 h at room temperature and saturated with a solution of 1% BSA in PBS for 1 h at room temperature. Whole blood was drawn from the inferior vena cava of mice anesthetized with a mixture of Imalgene (25 mg kg^−1^) and Rompun (10 mg kg^−1^) at 1 IU ml^−1^ and labeled with DIOC_6_ (2 μM) for 10 min at 37 °C. Using a syringe pump (PHD-2000, Harvard Apparatus), DIOC_6_-labeled whole blood was perfused through a collagen-coated microcapillary for 2 min at an arterial shear rate of 500 s^−1^. Thrombus formation was visualized with a ×40 oil-immersion objective for both fluorescence and transmitted light microscopy using the Zeiss Axio Observer microscope and recorded in real time (one frame every 20 s on 50 sections of 2 µm) with an ORCA camera (Carl Zeiss). Surface covered by platelets and three-dimensional thrombus volumes were calculated by thresholding using, respectively, ImageJ and Imaris.

### Platelet activation assays

For platelet aggregation and ATP release analysis, 300 µl of 2 × 10^8^ cells per ml washed platelets were stimulated in HEPES-Tyrode’s buffer (pH 7.38) with CRP (1 and 3 µg ml^−1^) or thrombin (0.1 and 0.3 IU ml^−1^) for 5 min at 37 °C under continuous stirring at 1,000 r.p.m. Aggregation measurements were performed using a Born lumi-aggregometer (Chrono-Log) alongside ATP release quantification by addition of 10 μl of Chronolume 1 min before stimulation. For P-selectin membrane exposure and αIIbβ3-integrin activation, 1 × 10^6^ platelets were stimulated with CRP (1 and 3 µg ml^−1^) or thrombin (0.1 and 0.3 IU ml^−1^) for 5 min at 37 °C under nonstirring conditions in the presence of 1 mM CaCl_2_ and PE-conjugated JON/A antibody. Platelets were further labeled with FITC-conjugated anti-mouse P-selectin for 30 min in the dark at room temperature before being analyzed with a BD LSRFortessa cytometer and BD FACSDiva software (BD Biosciences).

### Statistical analysis

All data are presented as mean ± s.e.m. and were obtained from ≥3 independent experiments with total sample numbers provided in the figure legends. Statistical significance was evaluated with Prism software (GraphPad), using a one-sample *t*-test with a theoretical mean of 1, two-tailed Student’s *t*-test, one-way ANOVA with Tukey’s multiple-comparisons test or two-way ANOVA followed by a Sidak’s multiple-comparisons test. Specific *P* values are indicated in the legends to figures. Significant differences are marked as follows: *P < 0.05, **P < 0.01, ***P < 0.001 and ****P < 0.0001.

### Reporting summary

Further information on research design is available in the Nature Research Reporting Summary linked to this article.

## Online content

Any methods, additional references, Nature Research reporting summaries, source data, extended data, supplementary information, acknowledgements, peer review information; details of author contributions and competing interests; and statements of data and code availability are available at 10.1038/s41589-022-01118-z.

## Supplementary information

Supplementary Information Supplementary Note (chemical synthesis of PITCOINs) and Supplementary Tables 1–4.

Reporting Summary

Supplementary Dataset Kinase profiling.

## Data Availability

Structural data were deposited in the PDB and are available under accession numbers 8A9I, 7Z74 and 7Z75. All other data are contained in the main manuscript, extended data and supplementary information. All materials and reagents are available from the corresponding authors. Source data are provided with this paper.

## Source data

Source Data Fig. 1 Statistical source data.
Source Data Fig. 3 Statistical source data.
Source Data Fig. 4 Statistical source data.
Source Data Fig. 5 Statistical source data.
Source Data Extended Data Fig. 1 Uncropped gel.
Source Data Extended Data Fig. 2 Statistical source data.
Source Data Extended Data Fig. 5 Statistical source data.
Source Data Extended Data Fig. 4 Statistical source data.
Source Data Extended Data Fig. 7 Statistical source data.
Source Data Extended Data Fig. 7 Uncropped western blot for Extended Data Fig. 7a.
