# Supplementary material for: Development of selective inhibitors of phosphatidylinositol 3-kinase C2α
Source: Nat Chem Biol. 2022 Sep 15;19(1):18–27. doi: 10.1038/s41589-022-01118-z (PMC7613998; doi:10.1038/s41589-022-01118-z)
Supplement: Supplementary file 1 — Supplementary Note (chemical synthesis of PITCOINs) and Supplementary Tables 1–4. [file 41589_2022_1118_MOESM1_ESM.pdf]

nature portfolio

https://doi.org/10.1038/s41589-022-01118-z

Supplementary information

Development of selective inhibitors of phosphatidylinositol 3-kinase C2α

In the format provided by the authors and unedited

Supplementary Note

Chemical synthesis of PITCOINs

All chemicals were purchased from commercial suppliers with the purity indicated and used as received unless otherwise specified. $CSCl_2$, from Sigma Aldrich > 82.5%; methyl 3-aminopyrazine-2-carboxylate from Sigma Aldrich 97%; phenethylamine from Sigma Aldrich >99%; 2-aminothiazole from Sigma Aldrich 97%; chloroacetyl chloride from Fluka 99%;2-bromo-1-(3-hydroxyphenyl)ethan-1-one from Carbosynth >96%; thiourea from Sigma Aldrich > 99%; 2-bromo-1-(3-nitrophenyl)ethan-1-one from Sigma Aldrich 97%; $SnCl_2$ from Sigma Aldrich 98%; methansulfonyl chloride, from Sigma Aldrich > 99.7%.

For all reactions, analytical grade solvents were used. All moisture sensitive reactions were carried out in oven-dried glassware (135 °C). ^{1}H-NMR and ^{13}C-NMR spectra were recorded either on AV 300 MHz or on AV 600 MHz from Bruker using DMSO (2.50 ppm) and $CHCl_3$ (7.26 ppm) as internal standard for ^{1}H-NMR spectra and DMSO-d_6 (39.5 ppm) or $CDCl_3$ (77.2 ppm) for ^{13}C-NMR spectra. Abbreviations used are: s = singlet, d = doublet, t = triplet, q = quartet, m = multiplet, br. s = broad singlet. Coupling constants (*J*) are expressed in Hz. NMR data were analyzed with MestReNova software. Mass spectra were obtained with two different spectrometers. LC-HRMS instrument: Agilent Technologies 6220 Accurate Mass TOF LC/MS linked to Agilent Technologies HPLC 1200 Series. LCMS instrument: Agilent Technologies 6120 Quadrupole LC/MS linked to Agilent HPLC 1290 Infinity. Purification of the compounds was performed by flash chromatography using an Isolera System from Biotage.

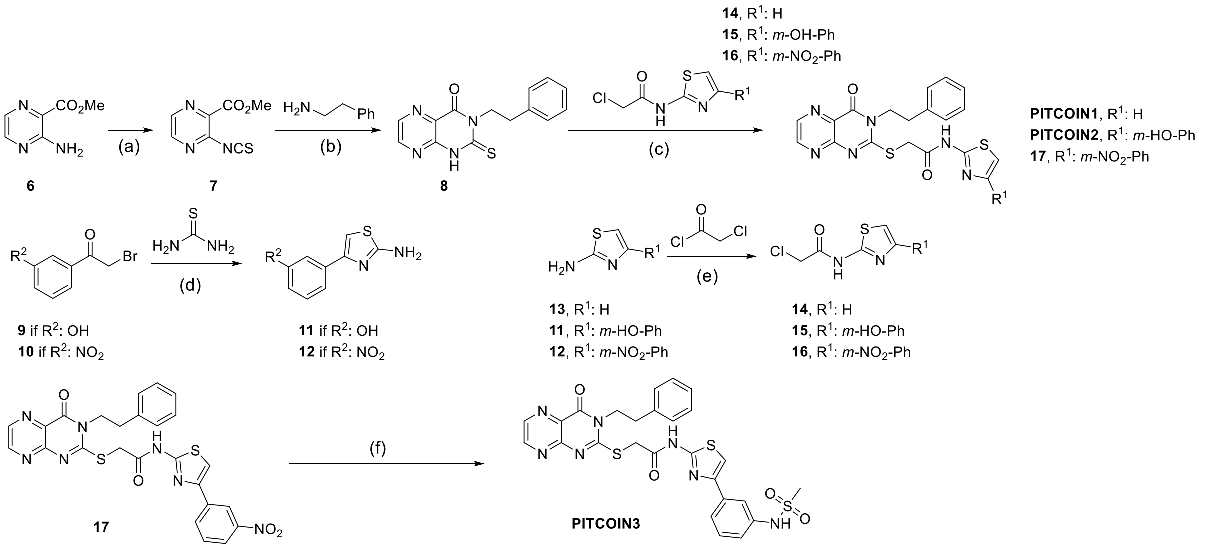

Synthesis scheme and reagents and conditions: (a) $CSCl_2$, Na_2CO_3, DCM, rt, 2 d, 98% yield; (b) 1,4-dioxane, 90°C, overnight, 98% yield; (c) chloroacetamide **14-16**, triethylamine, DMF, rt, overnight, 75-82% yield: (d) EtOH, reflux, 2 h, 73-91%; (e) triethylamine, DCM, 0°C to

rt, 15 min, 28-85% yield; (f) i. $SnCl_2$, EtOH, 2 h, 70°C, ii. $MeSO_2Cl$, triethylamine, DCM, 0°C to rt, 5 h, 18% yield over two steps.

Methyl 3-isothiocyanatopyrazine-2-carboxylate (**7**): To a solution of methyl 3-aminopyrazine-2-carboxylate **6** (1, 5.0 g, 32.7 mmol) in 60 mL dry DCM under nitrogen atmosphere was added a suspension of anhydrous Na_2CO_3 (13.2 g, 124.5 mmol, 3.8 eq.) in 30 mL anhydrous DCM. A solution of thiophosgene (4.6 g, 40.0 mmol, 1.2 eq.) in 30 mL dry DCM was added dropwise. The reaction mixture was stirred for 48 h at room temperature. After completion of the reaction as monitored by LCMS, inorganic salts were removed by filtration and washed with DCM. The filtrate was washed with water followed by a sat. $NaHCO_3$ solution and brine. The organic phase was dried over $MgSO_4$, filtered and concentrated under reduced pressure. The crude product was washed with an ethyl acetate/diisopropyl ether mixture (v/v, 1:20) to obtain product **7** as a solid (6.2 g, 32.0 mmol, 98%). ESI-MS (m/z): calcd for $[M + H]^+$ 196.0, found 196.1. ^{1}H NMR (300 MHz, $CDCl_3$) δ 8.58 (d, J = 2.4 Hz, 1H), 8.57 (d, J = 2.4 Hz, 1H), 4.06 (s, 3H).

3-Phenethyl-2-thioxo-2,3-dihydropteridin-4(1*H*)-one (**8**): To a solution of isothiocyanate **7** (1.08 g, 5.42 mmol, 1.0 eq.) in 1,4-dioxane (20 mL) was added phenethylamine (0.66 g, 5.42 mmol, 1.0 eq.). The mixture was stirred overnight at 90 °C. The mixture was cooled down to room temperature and the precipitated product was collected by filtration and washed twice a with ethyl acetate/diisopropyl ether (v/v, 1:1) mixture. Product **8** was obtained as a beige powder (1.52 g, 5.34 mmol, 98%). ESI-MS (m/z): calcd for $[M + H]^+$ 285.1, found 285.3. ^{1}H NMR (300 MHz, DMSO-d_6) δ 8.77 (d, J = 2.3 Hz, 1H), 8.65 (d, J = 2.3 Hz, 1H), 7.36 – 7.20 (m, 5H), 4.62 – 4.51 (m, 2H), 3.56 (s, 3H), 3.02 – 2.90 (m, 2H).

2-Chloro-*N*-(thiazol-2-yl)acetamide (**14**): To a solution of 2-aminothiazole **13** (1.0 g, 10.0 mmol, 1.0 eq.) and Et_3N (1.95 mL, 14.0 mmol, 1.4 eq.) in DCM (20 mL) was added a solution of chloroacetyl chloride (0.95 mL, 12.0 mmol, 1.2 eq.) in DCM (5 mL) dropwise at 0 °C under nitrogen atmosphere. The mixture was stirred for 15 min at 0 °C then 2 h at room temperature. The reaction was quenched with $NaHCO_3$ solution (20 mL) and the product was extracted with DCM (3x25 mL). Combined organic extracts were washed with brine, dried over $MgSO_4$ and concentrated under reduced pressure. The crude product was washed with a $MeOH/H_2O$ mixture (v/v, 1:1) to obtain the pure compound **14** (1.454 g, 8.23 mmol, 82%). ESI-MS (m/z):

calcd for $[M + H]^+$ 177.0, found 177.1. ^{1}H NMR (300 MHz, DMSO-d_6) δ 12.48 (s, 1H), 7.50 (d, *J* = 3.6 Hz, 1H), 7.27 (d, *J* = 3.6 Hz, 1H), 4.38 (s, 2H).

2-((4-Oxo-3-phenethyl-3,4-dihydropteridin-2-yl)thio)-*N*-(thiazol-2-yl)acetamide (PITCOIN1): To a solution of thiooxopteridinone **8** (100 mg, 0.35 mmol, 1.0 eq.) and chloroamide **14** (65 mg, 0.37 mmol, 1.05 eq.) in DMF (2 mL) was added Et_3N (0.98 mL, 0.70 mmol, 2.0 eq.) under nitrogen atmosphere. The reaction mixture was stirred overnight at room temperature. After the completion of the reaction (LCMS monitoring), the solvent was evaporated under reduced pressure. The residue was dissolved in DCM (20 mL) and water (15 mL). Phases were separated and aqueous phase was washed with DCM (15 mL) twice. Combined organic phases were dried $MgSO_4$, filtered and concentrated under reduced pressure. Further purification was done by automated column chromatography using silica gel as a stationary phase and a gradient of 0 – 25% MeOH in DCM as a mobile phase and the product was obtained as a colorless solid (112 mg, 0.26 mmol, 75% yield). ^{1}H NMR (300 MHz, DMSO-d_6) δ 12.56 (br s, 1H), 8.90 (d, *J* = 2.1 Hz, 1H), 8.76 (d, *J* = 2.1 Hz, 1H), 7.49 (d, *J* = 3.6 Hz, 1H), 7.41 – 7.24 (m, 5H), 7.22 (d, *J* = 3.6 Hz, 1H), 4.47 (s, 2H), 4.38 – 4.24 (m, 2H), 3.12 – 2.98 (m, 2H). ^{13}C NMR (151 MHz, DMSO-d_6) δ 165.90, 161.09, 160.02, 158.38, 152.91, 150.78, 144.22, 138.22, 138.05, 131.52, 129.18, 129.14, 127.27, 114.16, 46.67, 36.22, 33.46. HRMS (ESI) (m/z): $[M + H]^+$ calcd for $C_{19}H_{17}N_6O_2S_2$, 425.0849; found, 425.0846.

3-(2-Aminothiazol-4-yl)phenol (**11**): In a 100-mL two-neck round bottom flask equipped with a condenser was charged with a stirrer bar, 2-bromo-1-(3-hydroxyphenyl)ethan-1-one **9** (1.2 g, 5.58 mmol, 1.0 eq.) was dissolved in EtOH (35 mL). Afterwards, thiourea (424 mg, 5.58 mmol, 1.0 eq.) was added and the mixture was heated to 80 °C. After 2 h, it was cooled down and the solvent was evaporated under reduced pressure. The residue was dissolved in ethyl acetate (100 mL) and saturated $NaHCO_3$ solution (100 mL). The organic phase was separated and the aqueous phase was extracted twice with ethyl acetate (50 mL). Combined organic phases were dried over MgSO4, filtered, and concentrated under reduced pressure. Further purification was done by automated column chromatography using silica gel as a stationary phase and a gradient of 0 – 20% MeOH in DCM as a mobile phase and product **11** was obtained as an amorphous orange solid (980 mg, 5.10 mmol, 91% yield). ^{1}H NMR (300 MHz, DMSO-d_6) δ 9.37 (s, 1H), 7.21 (dp, *J* = 4.7, 1.4 Hz, 2H), 7.14 (t, *J* = 8.0 Hz, 1H), 7.02 (s, 2H), 6.90 (s, 1H), 6.65 (ddd, *J* = 7.8, 2.5, 1.3 Hz, 1H).

2-Chloro-*N*-(4-(3-hydroxyphenyl)thiazol-2-yl)acetamide (**15**): To a solution of amine **11** (980 mg, 3.01 mmol, 1.0 eq.) and Et_3N (0.924 mL, 6.63 mmol, 1.2 eq.) in DCM (15 mL), a solution of chloroacetyl chloride (0.446 mL, 5.61 mmol, 1.1 eq.) in DCM (5 mL) was added dropwise at 0 °C under nitrogen atmosphere. The mixture was stirred for 15 min at 0 °C then overnight at room temperature. The reaction was quenched with $NaHCO_3$ solution (20 mL) and the product was extracted with DCM (3x50 mL). The combined organic extracts were washed with brine, dried over $MgSO_4$ and concentrated under reduced pressure. The residue was washed with MeOH/H_2O (v/v, 1:1) to obtain product **15** as an amorphous orange solid (383 mg, 1.43 mmol, 28% yield). ^{1}H NMR (300 MHz, DMSO-d_6) δ 12.22 (s, 1H), 7.72 – 7.50 (m, 4H), 7.40 (s, 1H), 4.32 (s, 2H).

N-(4-(3-Hydroxyphenyl)thiazol-2-yl)-2-((4-oxo-3-phenethyl-3,4-dihydropteridin-2-yl)thio)acetamide (PITCOIN2): Chloroamide **15** (79 mg, 0.295 mmol, 1.05 eq.) was added to a solution of **8** (80 mg, 0.281 mmol) and Et_3N (78 µL, 0.563 mmol, 2.0 eq.) in DMF (2 mL) under nitrogen atmosphere. The reaction was stirred at room temperature overnight and, after completion of the reaction (monitored via LCMS), the solvent was evaporated under reduced pressure. The residue was dissolved in DCM (50 mL) and water (25 mL). Phases were separated and the aqueous phase was washed with DCM (25 mL) twice. Combined organic phases were dried $MgSO_4$, filtered and concentrated under reduced pressure. Further purification was done by automated column chromatography using silica gel as a stationary phase and a gradient of 0 – 25% MeOH in DCM as a mobile phase and the product was obtained as a colorless solid (118 mg, 0.228 mmol, 81% yield). ^{1}H NMR (300 MHz, DMSO-d_6) δ 12.72 (s, 1H), 9.52 (s, 1H), 8.89 (d, J = 2.1 Hz, 1H), 8.75 (d, J = 2.1 Hz, 1H), 7.54 (s, 1H), 7.38 – 7.25 (m, 7H), 7.22 (t, J = 7.9 Hz, 1H), 6.78 – 6.69 (m, 1H), 4.49 (s, 2H), 4.37 – 4.22 (m, 2H), 3.12 – 3.00 (m, 2H). ^{13}C NMR (75 MHz, DMSO-d_6) δ 167.67, 165.80, 160.63, 159.57, 157.67, 152.45, 150.33, 149.04, 143.76, 137.59, 135.51, 131.07, 129.74, 128.75, 128.70, 126.83, 116.56, 114.91, 112.64, 108.12, 46.24, 35.81, 33.01. HRMS (ESI) (m/z): $[M + H]^+$ calcd for $C_{25}H_{21}N_6O_3S_2$, 516.1038; found, 516.1271.

4-(3-Nitrophenyl)thiazol-2-amine (**12**): In a 100-mL two-neck round bottom flask equipped with a condenser was charged with a stirrer bar, 2-bromo-1-(3-nitrophenyl)ethan-1-one **10** (1.0 g, 4.10 mmol, 1.0 eq.), was dissolved in EtOH (35 mL). Afterwards, thiourea (312 mg, 4.10 mmol, 1.0 eq.) was added and the mixture was heated to 80 °C. After 2 h, it was cooled down and the solvent was evaporated under reduced pressure. The residue was dissolved in ethyl

acetate (100 mL) and saturated $NaHCO_3$ solution (100 mL). The organic phase was separated and the aqueous phase was extracted twice with ethyl acetate (50 mL). Combined organic phases were dried over $MgSO_4$, filtered, and concentrated under reduced pressure. Further purification was done by column chromatography using silica gel as a stationary phase and a gradient of 0 – 20% MeOH in DCM as a mobile phase and product **12** was obtained as an amorphous orange solid (73% mg, 3.01 mmol, 73% yield). ^{1}H NMR (300 MHz, DMSO-d_6) δ 8.58 (t, *J* = 2.0 Hz, 1H), 8.26 – 8.19 (m, 2H), 7.75 (t, *J* = 8.0 Hz, 1H), 7.49 (s, 1H).

2-Chloro-*N*-(4-(3-nitrophenyl)thiazol-2-yl)acetamide (**16**): To a solution of amine **12** (672 mg, 3.01 mmol, 1.0 eq.) and Et_3N (0.588 mL, 4.21 mmol, 1.4 eq.) in DCM (15 mL), a solution of chloroacetyl chloride (0.290 mL, 3.65 mmol, 1.2 eq.) in DCM (5 mL) was added dropwise at 0 °C under nitrogen atmosphere. The mixture was stirred for 15 min at 0 °C then overnight at room temperature. The reaction was quenched with $NaHCO_3$ solution (20 mL) and the product was extracted with DCM (3x50 mL). Combined organic extracts were washed with brine, dried over $MgSO_4$ and concentrated under reduced pressure. The residue was washed with MeOH/H_2O (v/v, 1:1) to obtain product **16** as amorphous orange solid (767 mg, 2.58 mmol, 85% yield). ^{1}H NMR (300 MHz, DMSO-d_6) δ 12.78 (s, 1H), 8.70 (s, 1H), 8.34 (d, *J* = 7.4 Hz, 1H), 8.19 (s, 1H), 8.01 (s, 1H), 7.89 – 7.58 (m, 1H), 4.40 (d, *J* = 21.4 Hz, 2H).

N-(4-(3-Nitrophenyl)thiazol-2-yl)-2-((4-oxo-3-phenethyl-3,4-dihydropteridin-2-yl)thio)acetamide (**17**): Chloroamide **16** (385 mg, 1.29 mmol, 1.05 eq.) was added to a solution of **8** (350 mg, 1.23 mmol) and Et_3N (0.377 mL, 2.71 mmol, 2.2 eq.) in DMF (15 mL) under nitrogen atmosphere. The reaction was stirred at room temperature overnight and, after completion of the reaction (monitored via LCMS), the solvent was evaporated under reduced pressure. The residue was dissolved in DCM (100 mL) and water (50 mL). Phases were separated and the aqueous phase was washed with DCM (50 mL) twice. Combined organic phases were dried $MgSO_4$, filtered and concentrated under reduced pressure. Further purification was done by column chromatography using silica gel as a stationary phase and a gradient of 0 – 25% MeOH in DCM as a mobile phase and product **17** was obtained as a colorless solid (554 mg, 1.02 mmol, 82% yield). ^{1}H NMR (300 MHz, DMSO-d_6) δ 12.87 (br s, 1H), 8.89 (d, *J* = 2.1 Hz, 1H), 8.80 – 8.72 (m, 2H), 8.42 – 8.32 (m, 1H), 8.24 – 8.14 (m, 1H), 7.96 (d, *J* = 5.4 Hz, 1H), 7.75 (t, *J* = 8.0 Hz, 1H), 7.42 – 7.22 (m, 5H), 4.50 (s, 2H), 4.37 – 4.25 (m, 2H), 3.13 – 3.01 (m, 2H).

N-(4-(3-(Methylsulfonamido)phenyl)thiazol-2-yl)-2-((4-oxo-3-phenethyl-3,4-dihydropteridin-2-yl)thio)acetamide (PITCOIN3): To a solution of **17** (550 mg, 1.01 mmol) in ethanol (10 mL), $SnCl_2$ (956 mg, 5.04 mmol, 5.0 eq.) was added at room temperature. The mixture was heated to 70 °C. After 2h, it was cooled to ambient temperature and then was poured on crushed ice and quenched with saturated $NaHCO_3$ solution. The mixture was extracted with DCM (80 mL) three times. Combined extracts were washed with brine, dried over $MgSO_4$ and concentrated under reduced pressure. The crude product was employed for the next reaction without further purification. Methansulfonyl chloride (102 µL, 1.01 mmol, 1 eq.) was added dropwise at 0 °C to a solution the crude aniline, Et_3N (281 µL, 7.40 mmol, 2.0 eq.) in DCM (5 mL). The reaction was stirred at 0 °C for 5 h. After completion of the reaction (monitored via LCMS). The mixture was extracted in DCM (50 mL) and with a saturated water solution of Na_2CO_3 (25 mL). Phases were separated and the aqueous phase was washed with DCM (50 mL) twice. Combined organic phases were dried $MgSO_4$, filtered. Further purification was done by column chromatography using silica gel as a stationary phase and a gradient of 0 – 25% MeOH in DCM as a mobile phase and the product was obtained as a colorless solid (108 mg, 0.182 mmol, 18% yield). ^{1}H NMR (300 MHz, DMSO-d_6) δ 12.80 (br s, 1H), 8.89 (br s, 1H), 8.75 (br s, 1H), 7.94 (s, 1H), 7.85 (d, J = 7.6 Hz, 1H), 7.73 (s, 1H), 7.57 – 7.14 (m, 7H), 4.49 (s, 2H), 4.31 (t, J = 8.1 Hz, 2H), 3.07 (t, J = 8.1 Hz, 2H), 2.98 (s, 3H). ^{13}C NMR (151 MHz, DMSO-d_6) δ 166.36, 161.08, 160.02, 158.37, 152.91, 150.78, 148.96, 144.22, 139.37, 138.05, 135.79, 131.53, 130.15, 129.19, 129.15, 127.28, 121.83, 119.95, 117.58, 109.33, 46.68, 40.56, 36.22, 33.47. HRMS (ESI) (m/z): $[M + H]^+$ calcd for $C_{26}H_{24}N_7O_4S_3$, 593.0974; found, 593.1021.

Supplementary Table 1 | Small molecule screening data

Category	Parameter	Description
Assay	Type of assay	ADP-Glo kinase assay (Promega)
	Target	PI3KC2α
	Primary measurement	Luminescence
	Key reagents	Liver PI (Avanti, 84002)
	Assay protocol	Kinase reaction: 40 μM ATP, 200 μM Liver PI, 10 ng/μl purified PI3KC2α (total volume, 5ul) for 20 mins, ADP-Glo reaction: 5 μl for 40 mins at RT, kinase detection: 10 μl for 20 min. All reactions were performed at room temperature. The buffer used was: 5 mM Hepes pH 7.2, 25 mM KCl, 2.5 mM MgOAc, 150 mM KGlu, 10 μM $CaCl_2$, 0.2 % CHAPS, 1 mM DTT, 5 mM $MgCl_2$.
	Additional comments	
Library	Library size	37,664 chemical compounds
	Library composition	Small molecule diversity set library (33,088) and compounds donated from academic groups (4,576)
	Source	FMP compound library
	Additional comments	
Screen	Format	384-well white small-volume microtiter plate
	Concentration(s) tested	30 μM
	Plate controls	Positive control: kinase reaction at 23rd column<br>Negative control: kinase reaction without PI at 24th column
	Reagent/ compound dispensing system	Biotek dispenser for kinase stock solution<br>BeckmanFX Workstation equipped with V&P Scientific FP1 pin tool for compound transfer.<br>Tecan Evo Workstation with integrated SafireII plate reader for lipid/ATP stock solution, ADP-Glo reagent, kinase detection reagent addition and luminescence reading.
	Detection instrument and software	Tecan SafireII plate reader and Magellan^{TM} software
	Assay validation/QC	Initial assay validation was performed using PI3 kinase inhibitor Wortmannin to verify concentration-dependent detection of inhibitor activity. During HTS, on each plate the Z'-factor was calculated using the positive and negative control columns, and for each sample the Z-Score was calculated. The plate data was visualized with scatterplots and heatmaps to allow visual detection of experimental artifacts (see "platemaps" in appendix). On the whole-screen level, the distribution of all obtained Z-scores was plotted to verify a normal-distribution like behavior of the data (see "PrimaryScreeningReport" in appendix).
	Correction factors	Robust estimators for mean (median) and stddev (median absolute deviation * 1.48258) were used
	Normalization	Data was normalized for each single measured plate ("Z-score": deviation of the signal towards the mean of all samples, in units of stddev, "rel_activ": activity relative to the plate controls, "Median acitivity": activity relative to the median signal of all samples on a plate). No normalization algorithms for correcting positional effects or instrument artifacts were used.
	Additional comments	
Post-HTS analysis	Hit criteria	Z-score < -3
	Hit rate	1.08%
	Additional assay(s)	IC50 validation of selected 352 compounds sourced from 407 HTS positives; selected hits profiled in a lipid kinase assay panel (#22) at Thermo Fisher Scientific
	Confirmation of hit purity and structure	Yes (LC-HRMS, 1H-NMR and resynthesis of selected hits)
	Additional comments	

Supplementary Table 2 | IC_{50} values of PITCOIN1-3 against PI3K family members.

PI3Ks	PITCOIN1 (IC_{50})	PITCOIN2 (IC_{50})	PITCOIN3 (IC_{50})
PI3KC2α	95 nM	121 nM	126 nM
PI3KC2β	5,700 nM	1,399 nM	>10,000
PI3KC2γ	860 nM	>10,000	>10,000
PI3Ks (α, β, γ, δ)	>10,000	>10,000	>10,000
VPS34	>10,000	>10,000	>10,000

Supplementary Table 3 | Data collection and refinement statistics

	PI3KC2α core (PITCOIN1)	PI3KC2α core (PITCOIN2)	PI3KC2α core (PITCOIN3)
**Data collection**			
Space group	$P2_12_12_1$	$P2_12_12_1$	$P2_12_12_1$
Cell dimensions			
a, *b*, *c* (Å)	56.2, 133.3, 152.5	56.3, 135.2, 151.9	56.1, 134.4, 152.7
α, β, γ (°)	90.0, 90.0, 90.0	90.0, 90.0, 90.0	90.0, 90.0, 90.0
Resolution (Å)	47.49 – 2.87 (2.97 – 2.87)	43.26 – 2.50 (2.59 – 2.50)	49.01– 2.60 (2.69 – 2.60)
R_{merge}	0.14 (1.92)	0.14 (3.81)	0.11 (3.07)
$I / \sigma I$	9.94 (0.70)	9.02 (0.36)	9.98 (0.50)
Completeness (%)	98.7 (99.7)	99.7 (99.7)	98.9 (95.7)
Redundancy	4.0 (4.1)	5.5 (5.5)	5.4 (5.3)
**Refinement**			
Resolution (Å)	2.87	2.50	2.60
No. reflections	26636	40881	36215
R_{work} / R_{free}	22.5/ 26.3	22.9 / 26.9	22.3 / 26.7
No. atoms			
Protein	6521	6485	6547
Ligand/ion	42	56	49
Water	65	74	62
B-factors			
Protein	89.55	86.89	94.1
Ligand/ion	96.41	96.86	135.0
Water	58.30	78.84	77.3
R.m.s. deviations			
Bond lengths (Å)	0.009	0.007	0.013
Bond angles (°)	1.55	1.1	1.88

*1 crystal for each structure was used for data collection and structure determination.
*Values in parentheses are for highest-resolution shell.

Supplementary Table 4 | Oligonucleotides

Oligonucleotides	SOURCE	IDENTIFIER
Scrambled siRNA	Sigma	MISSION Universal Negative Control #1
PI3KC2α siRNA<br>GCACAAACCCAGGCUAUUU	Posor, et. al. 2013	
Human PI3KC2a376_KasI_forward<br>GATCGGCGCCGTACAGAATGAGGAGATGGCAGCTTTTTG	This paper	
Human PI3KC2a1682_XbaI&NotI_reverse<br>GATCGCGGCCGCTCTAGACTATGCCGCAGTCAGCTGATACC	This paper	
musPI3KC2a377_KasI_forward<br>GATCGGCGCCGTACAGAATGACGAGGTGGCAGCTTTTTG	Lo et. al. (2022) *Nat Struct Mol Biol* 29, 218-228.	
musPI3KC2a1400_XbaI_reverse<br>GATCTCTAGATTAGCCAGAAAAACGTAGCTGAGCAAGGTTATG	Lo et. al. (2022) *Nat Struct Mol Biol* 29, 218-228.	
MusPI3KC2a_533-544/GSGS_forward<br>AGAACCGTTTAAATCCACAGGTGCTTCATCATC	Lo et. al. (2022) *Nat Struct Mol Biol* 29, 218-228.	
MusPI3KC2a_533-544/GSGS_reverse<br>GGCTCTGTCATGACAAGACACCCTGTTGAAG	Lo et. al. (2022) *Nat Struct Mol Biol* 29, 218-228.	
musPI3KC2a550-665/SAGAGSGA_forward<br>CGCACCCGCAGAGTGTCTTGTCATGACAGAGCCAG	Lo et. al. (2022) *Nat Struct Mol Biol* 29, 218-228.	
musPI3KC2a550-665/SAGAGSGA _reverse<br>GGTAGTGCCGGTTCTACAGGCTGTCCCCGAGG	Lo et. al. (2022) *Nat Struct Mol Biol* 29, 218-228.	
