# Supplementary figures and images for: Development of selective inhibitors of phosphatidylinositol 3-kinase C2α
Source: Nat Chem Biol. 2022 Sep 15;19(1):18–27. doi: 10.1038/s41589-022-01118-z (PMC7613998; doi:10.1038/s41589-022-01118-z)

SEC fractions-  
PI3KC2a deltaN

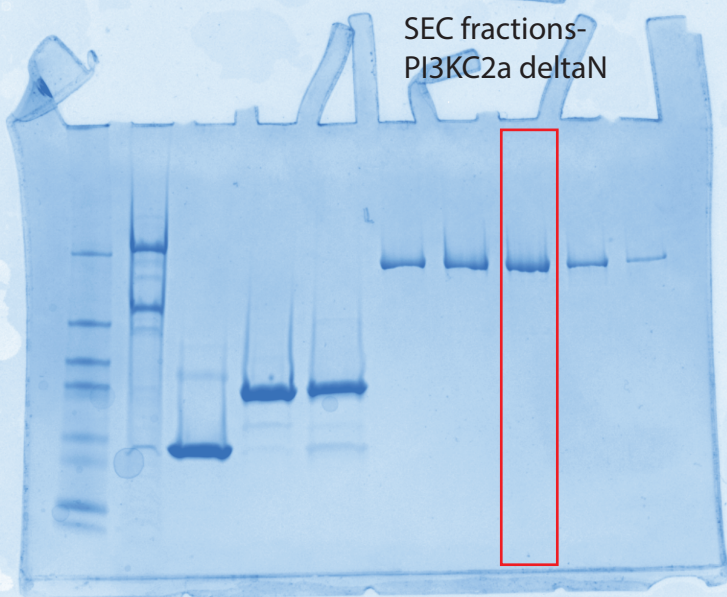

sample before  
crystallization  
PI3KC2a core

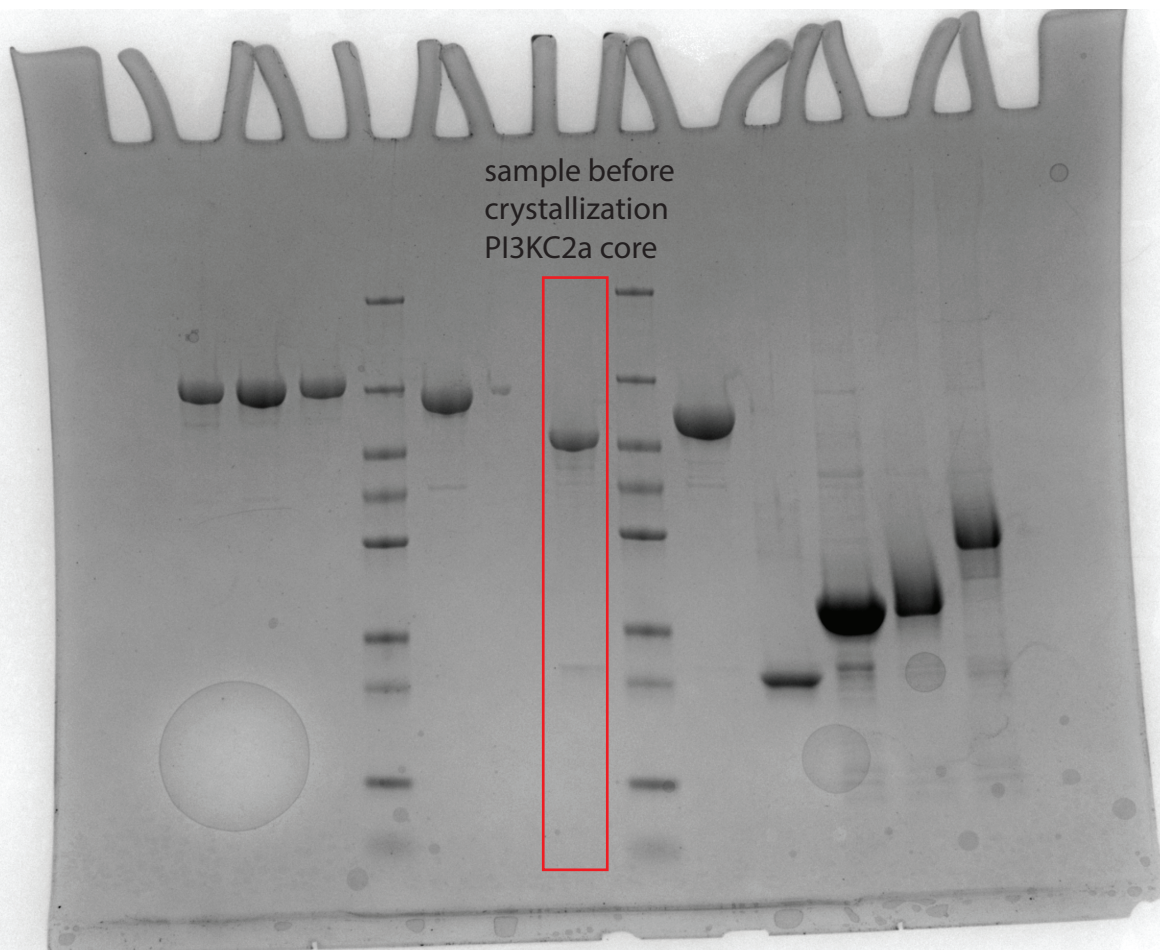

Supplement: Source Data Extended Data Fig. 1 — Uncropped gel. [file 41589_2022_1118_MOESM8_ESM.pdf]

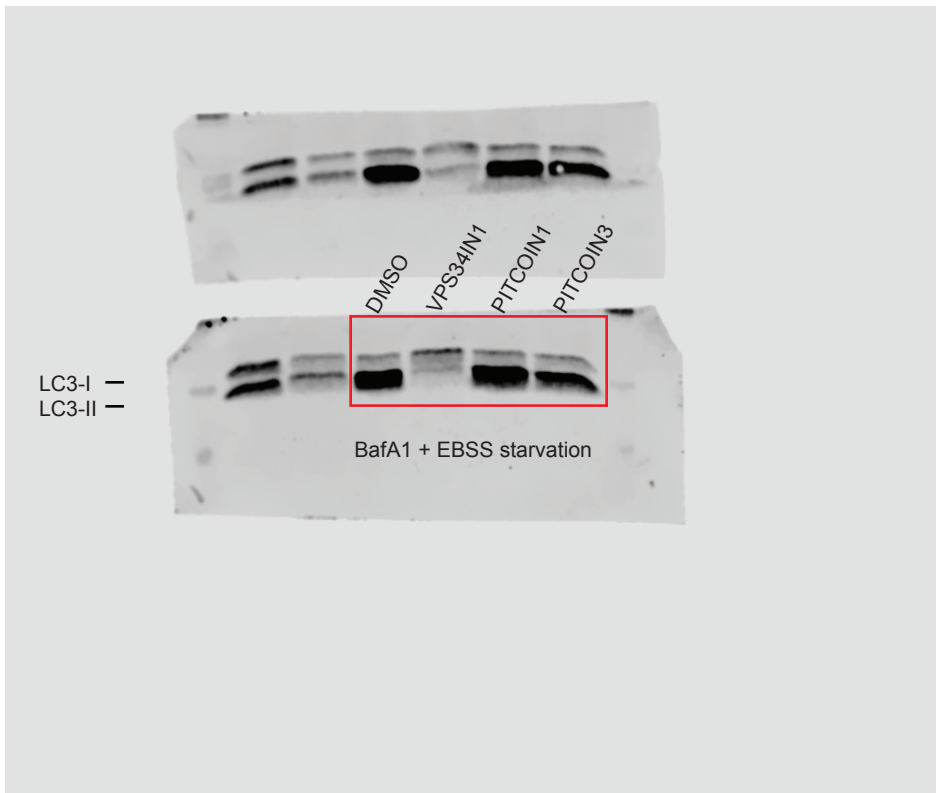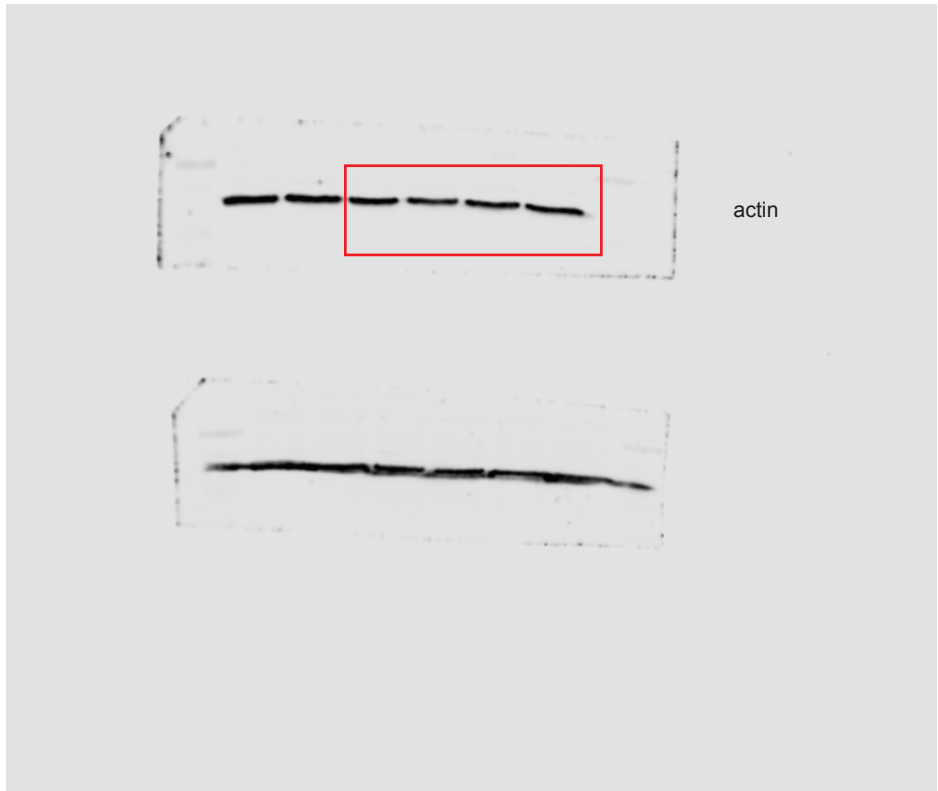

Supplement: Source Data Extended Data Fig. 7 — Uncropped western blot for Extended Data Fig. 7a. [file 41589_2022_1118_MOESM13_ESM.pdf]
